# Refining Bayesian hierarchical MPT modeling: Integrating prior knowledge and ordinal expectations

**DOI:** 10.3758/s13428-024-02370-y

**Published:** 2024-04-16

**Authors:** Alexandra Sarafoglou, Beatrice G. Kuhlmann, Frederik Aust, Julia M. Haaf

**Affiliations:** 1 Department of Psychology, University of Amsterdam, Nieuwe Achtergracht 129B, 1001 NK Amsterdam, The Netherlands; 2 Department of Psychology, School of Social Sciences, University of Mannheim, Mannheim, Germany

**Keywords:** Bayes factors, MPT models, Inequality constraints

## Abstract

Multinomial processing tree (MPT) models are a broad class of statistical models used to test sophisticated psychological theories. The research questions derived from these theories often go beyond simple condition effects on parameters and involve ordinal expectations (e.g., the same-direction effect on the memory parameter is stronger in one experimental condition than another) or disordinal expectations (e.g., the effect reverses in one experimental condition). Here, we argue that by refining common modeling practices, Bayesian hierarchical models are well suited to estimate and test these expectations. Concretely, we show that the default priors proposed in the literature lead to nonsensical predictions for individuals and the population distribution, leading to problems not only in model comparison but also in parameter estimation. Rather than relying on these priors, we argue that MPT modelers should determine priors that are consistent with their theoretical knowledge. In addition, we demonstrate how Bayesian model comparison may be used to test ordinal and disordinal interactions by means of Bayes factors. We apply the techniques discussed to empirical data from Bell et al. *Journal of Experimental Psychology: Learning, Memory, and Cognition*, *41*, 456–472 (2015).

**Supplementary Information:**

The online version contains supplementary material available at 10.3758/s13428-024-02370-y.

## Introduction

Multinomial processing tree (MPT) models are a broad class of statistical models to estimate probabilities of latent cognitive processes underlying observed behavior (Riefer & Batchelder, 1988; Batchelder & Riefer, 1999). In psychology, MPT models are used to test sophisticated theories of memory, judgement and decision-making, and reasoning (for a review on the literature, see Erdfelder et al., 2009). These sophisticated theories make predictions for data from experimental tasks and in many cases these predictions are very specific. For instance, based on aging theories, researchers may predict that memory retrieval is more affected by an experimental manipulation for older adults than for younger adults. This prediction is a specific ordinal interaction, however, specifying a statistical model for this prediction is not entirely trivial. We argue that while hierarchical Bayesian MPT modeling is well suited to testing these nontrivial predictions, current Bayesian MPT modeling practices leave room for refinement. In particular, we will argue that Bayesian model comparison may be used to easily test specific ordinal interactions, and that the most commonly used priors (Klauer, 2010; Matzke et al., 2015) are simultaneously too vague and too informative for most applications. We will elaborate these arguments using a MPT model that instantiates a psychological theory of source memory, namely the 2-High-Threshold Source Monitoring (2HTSM) model.

Source memory captures the ability to remember contextual details that accompanied a piece of learned information (Johnson et al., 1993), such as the face of the person describing directions to the bus stop. A typical paradigm to study source memory consists of a study phase and a test phase (e.g., Batchelder and Riefer, 1990; Johnson et al., 1993). In the study phase, participants are presented with items stemming from one of several sources (e.g., words spoken by a female or male voice). In the test phase, participants are presented with the learned items again along with new items. Participants must then decide for each item whether it is a new item or has been presented before, and if so, by what source.

While the source memory paradigm is fairly simple, multiple psychological processes are most likely at play. Suppose someone correctly identified an item as old, and also correctly identified the source of the item. Then this correct identification could be due to actual mnemonic information about the item and the source, or it could be due to guessing. Note that two guessing processes – about the item and the source – might be at play here. The interlocking of guessing and memory processes on several levels is the reason why MPT modeling is so popular in source memory research (Erdfelder et al., 2009).

Traditionally, MPT models are specified in the classical frequentist framework. However, Bayesian modeling has increasingly become the tool of choice in the MPT literature, since it facilitates the specification of complex models. For instance, it is reasonable to assume that cognitive processes such as guessing vary across individuals, an assumption that can easily be implemented in the Bayesian framework by extending MPT models as hierarchical models (Rouder & Lu, 2005; Rouder et al., 2017; see Schmidt et al., 2022 for a tutorial on Bayesian MPT models).

**Fig. 1:**
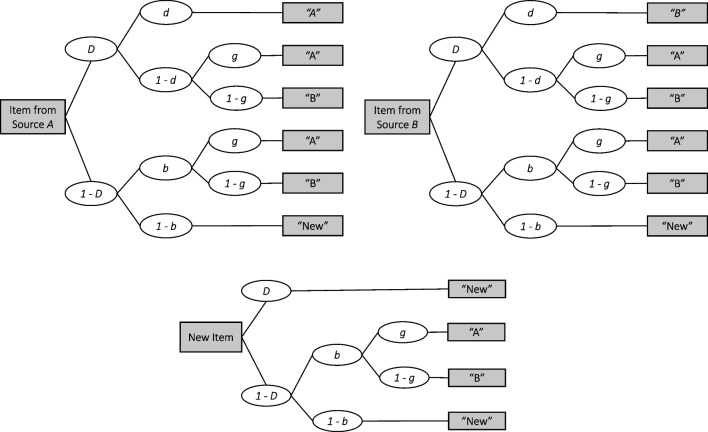
Tree architecture for a paradigm of the 2HTSM model in its most parsimonious version. In a source memory task, participants are presented with items that they have previously learned and that either stem from source A, source B (top two trees), or are new items (bottom tree). They then have to distinguish previously learned items from new items and must decide for the previously learned items from which source they originate. The 2HTSM model assumes that participants responses’ depend on four cognitive processes: item memory *D*, source memory *d*, item guessing *b*, and source guessing *g*

Within the Bayesian framework, the specification of an MPT model requires three steps. The first step is routine for most MPT modelers: specifying the MPT model equations within one experimental condition. These equations formalize assumptions about the cascade of distinct cognitive processes that contribute to the behavior of interest. Each process is associated with a model parameter that controls the probability with which the process is engaged. These equations are often communicated as tree-like diagrams, as illustrated in Fig. 1. The second step is to specify the statistical model on parameters within one experimental condition. This step includes the formulation of general model assumptions, such as how variability of items or participants are modeled, but also the specification of adequate prior distributions on the MPT parameters. The third step concerns the expected effects on the model parameters across experimental conditions. In most cases, this step corresponds to the specification of the main research question as competing statistical models. Here, we will focus on steps two and three.

### Specifying informative prior distributions

The choice of prior distributions for model parameters often sparks a philosophical debate between objective Bayesians and subjective Bayesians (see Berger (2004) and Aczel et al. (2020) for perspectives on this topic). Objective Bayesians construct prior distributions such that the prior or resulting inference has mathematical properties deemed desirable. Objective prior distributions may be chosen for their invariance under model reparameterization (Bernardo, 1979), for assigning in the absence of other information equal probability to all possible parameter values (Keynes, 1921, i.e., indifference principle), or for maximizing uncertainty while satisfying model constraints (Jaynes, 2016; Shannon, 1948). Common to all objective priors is that they do not incorporate prior domain knowledge and yield results that are informed largely by the likelihood function and align closely with results from frequentist statistics – they let the data speak for themselves. What is often disregarded, however, is that when priors do not align with theoretical expectations, it may result in nonsensical predictions and thus compromise model comparison using Bayes factors, which relies on the predictive accuracy of the models under consideration (Lee & Vanpaemel, 2018, but see Moreno & Pericchi, 2014.

Following objective Bayesian philosophy, the prior distributions for hierarchical latent-trait MPT models proposed by Matzke et al. (2015) (based on Klauer, 2010) and implemented in the R package TreeBUGS (Heck et al., 2018) were intentionally designed not to embody any psychological theory, resulting in diffuse distributions with a broad range on the latent space. However, these seemingly vague and uninformative prior distributions on the group-level have serious consequences about individual-level parameter values and response rates: they imply highly informative and nonsensical predictions.

Using the 2HTSM model (Bayen et al., 1996) we will demonstrate that prior predictions made from models with diffuse priors on the group-level parameters – even if they feature sophisticated model equations – are at odds with theoretical expectations. We argue that these priors are therefore, despite their wide use in the MPT literature, inappropriate for testing theory, and may under some conditions even be problematic for parameter estimation.^1^ Note that in the following, when discussing Matzke–Klauer priors, we refer to their most constrained version, that is, the default implementation in TreeBUGS.

To ensure that model predictions align with theoretical expectations, we therefore advocate for more subjectivity in Bayesian inference. Subjective Bayesians view the construction of prior distributions implemented in step two as akin to formulating the model equation, an integral part of model development. Since the model parameters correspond to psychological variables, it is crucial for their prior distributions to capture values that are permissible, likely, unlikely, or non-permissible based on the underlying theory (Lee & Vanpaemel, 2018).

#### Possible concerns in the construction of informative priors

Researchers are often reluctant to utilize their own expertise in determining prior distributions. They may choose default priors for convenience, or may be unaware of how uninformative priors can impact their models. They may fear that informative priors could spoil their model evaluation (Kass & Raftery, 1995), or invite criticism from other researchers. For this reason, researchers may tend to adopt the default priors discussed in the literature and implemented in software without any adjustments, assuming that less information – and thus subjectivity – will lead to the most valid results. Rather than viewing prior specification as a burden or a necessary evil, many Bayesians have been advocating a change in perspective: when researchers work with quantitatively instantiated theories – which MPT models undoubtedly are – prior distributions along with model equations, are an opportunity to fully describe all aspects captured by theory (e.g., Dienes, 2011; Rouder et al., 2016; Vanpaemel, 2010; Vanpaemel & Lee, 2012).

We believe that MPT modelers possess the necessary expertise to refine their models in line with domain knowledge, which is evident in their thoughtful considerations of model parameters and their interaction when constructing MPT model equations. Importantly, their expertise encompasses expectations about typical data patterns and the heterogeneity of the sample. Researchers can thus construct informative priors based on their own expertise, prior literature, consensus knowledge through expert elicitation or systematic parameter reviews (Vanpaemel, 2010; Lee, 2018; Lee & Vanpaemel, 2018; Stefan et al., 2020; Tran et al., 2021; Stefan et al., 2022; O’Hagan et al., 2006). Additionally, MPT models are typically based on a solid empirical foundation that offers valuable insights into the shape of parameter distributions, such as expected values and variances in memory parameters across different age groups, or (dis)ordinal interactions between experimental conditions. Hence, empirical data can also partially inform the choice of priors (e.g., Gu et al., 2018; O’Hagan, 1995).

### Model comparison for Bayesian hierarchical MPT models

Theories that are tested with MPT models are often quite sophisticated, thus requiring complex experimental designs. In turn, MPT models need to be specified to account for a rich set of predictions of experimental effects on cognitive parameters. These predictions often concern ordinal expectations (e.g., bias is higher in one condition than in another) or disordinal expectations (e.g., the effect reverses in one experimental condition) of multiple interaction effects.

In step three of model specification, these predictions are implemented. Traditionally, competing models are implemented in the frequentist framework, and an encompassing model is tested against a model with constraints on parameters across experimental conditions. In this framework, ordinal constraints are implemented by changing the likelihood of the model, for instance, by reparameterization (Knapp & Batchelder, 2004; Klauer et al., 2015; Kuhlmann et al., 2019). Reparametrization is used to reformulate ordinal constraints for different experimental conditions such that an effect in one condition is represented by a shrinkage factor of another condition. For instance, it is equivalent to reformulate the ordinal constraint $g_1 < g_2$ on guessing parameters in two conditions as $g_1 = a \times g_2$. The ordering of the parameters is implemented by restricting the shrinkage factor *a* to values between 0 and 1. Reparametrization provides an elegant solution to the problem of testing ordinal hypotheses, and ensures that the effects are in the same direction across scale transformations, but also complicates the formulation of the model equations and the interpretation of the MPT parameters, especially when the ordinal constraint spans multiple experimental conditions.

More recently, Bayesian model comparison using Bayes factors has also gained traction in MPT modeling, mainly due to computational progress (e.g., Gronau et al., 2019). However, Bayes factor model comparison is not yet common practice, in part because it is challenging to specify MPT models that correspond to specific hypotheses and to evaluate to what extent they are supported by the data. The success of this endeavor depends entirely on how well the researcher succeeds in building their mathematical model. Here, we provide a simple solution to incorporate a set of equality and ordinal constraints on parameters across experimental conditions, and to test these constraints using Bayes factors.

The structure of the paper is as follows. First, we introduce the Bayesian implementation of the hierarchical 2HTSM model. Second, we describe how predictions from the 2HTSM model aid model specification, including the selection of appropriate prior distributions. Third, we show how to compare MPT models by means of the Bayes factor. The models under consideration include predictions about the order of the size of the MPT parameters across experimental conditions, that is, ordinal and disordinal interactions. We show that instead of reparameterizing the models to implement the constraints (i.e., modify their likelihood) the constraints can be implemented directly in the prior distributions. Finally, we illustrate our methods with one case study using empirical data from Bell et al. (2015).

### Availability of data and code

Readers can access the R code to reproduce all analyses (including the prior simulation study and the creation of all figures in our OSF folder at: https://osf.io/bpgc5/. The OSF folder also features a TreeBUGS implementation of our case study with similar but not exactly identical priors. The data needed to run the reanalysis from Bell et al. (2015) were kindly provided by the authors and can be accessed in our OSF folder.

## The two-high threshold model for source monitoring

We start with the first step MPT modelers go through to express their theory by a mathematical model. This step concerns the formulation of the model equation and the formulation of general model assumptions. In its most parsimonious version (submodel 4), the 2HTSM model proposed by Bayen et al. (1996) assumes four independent cognitive processes to contribute to a response in a source memory paradigm. According to the model, participants need to cross two thresholds to be able to fully remember a previously presented (old) item and its source. If participants cross the first threshold, they remember an item as old. The probability of crossing this item recognition threshold is represented by the parameter *D*. Participants who also cross the second threshold remember an item’s source. The probability of crossing this source recognition threshold is represented by parameter *d*. For old items, if either of these thresholds is not crossed, guessing processes will partially or fully determine the response behavior. Parameter *b* describes the probability of guessing that an item was part of the study list (item guessing), and parameter *g* describes the probability of guessing that the item stems from a particular source (source guessing). For new items, there is a third threshold for distractor detection that is assumed to be crossed with the same probability as for item recognition (i.e., parameter *D*); if this threshold is not crossed, the response behavior is again determined by guessing processes. In Fig. 1, we illustrate the tree architecture of the 2HTSM model. Note, however, that the architecture is typically adapted to the specific experimental paradigms used in a study (e.g., using the graphical model builder by Moshagen, 2010).

### Specification of the statistical model

The second step of model specification involves the specification of the statistical model within one experimental condition. This specification concerns the treatment of participants and items. Arguably, when experimental materials are standardized and validated in pilot studies, item heterogeneity can be well controlled, justifying aggregation across items. The assumption of homogeneity of individuals on the other hand is more problematic (e.g., Rouder and Lu, 2005; Rouder et al., 2008; Webb and Lee, 2004, but see Matzke et al., 2015 and Smith and Batchelder, 2008). Since MPT parameters reflect psychological processes (e.g., memory performance), which depend on individual participant characteristics (e.g., age, response biases, stereotypes), it is useful to allow for individual differences in the model. Within the Bayesian framework, two model classes have been established to account for individual differences, the beta-MPT model (Smith & Batchelder, 2010), and the latent-trait model (Klauer, 2010).

The beta-MPT model assumes that individual-level MPT parameters stem from independent group-level beta distributions. As MPT parameters are modeled in the probability space on the individual level and the group level, prior selection is intuitive. In contrast, the latent-trait model transforms the parameter space to a latent continuous space. The benefit of a latent continuous space is that intuitions from generalized linear models are appropriate here, simplifying the development of regression models on specific parameters. In addition, the latent-trait model allows for the specification of a covariance matrix that models the correlation across participants and allows for more hierarchical shrinkage. Since hierarchical shrinkage is necessary to avoid overestimating individual differences, we will focus on the latent-trait model in the remainder of the paper. The full mathematical specification of the latent-trait models discussed in this manuscript are provided in Appendix A.

In the latent-trait approach, assuming item homogeneity, participant responses are aggregated over items in each experimental condition. The category frequencies are assumed to follow a multinomial distribution with the underlying category probabilities resulting from the MPT model equation. In this model, all parameters are probit-transformed to a latent space. In this unbounded (latent) parameter space, the individual differences in the MPT parameters are then modeled. Specifically, it is assumed that the transformed parameters are normally distributed and may be correlated with other parameters (i.e., transformed parameters follow a multivariate normal distribution). The means of the multivariate distribution represent group-level parameters. The variance–covariance matrix determines the magnitude of individuals’ deviations around said group-level parameters and their correlation, thus determining the extent to which individuals can differ from one another. To capture both within-subject dynamics and independent between-subject effects, the relationships between model parameters for each individual were modeled using a covariance structure, while between-subject effects were modeled independently.

### The problem with default MPT priors

After establishing the model equation and the statistical model, we now turn to determining adequate prior distributions for the model priors. Priors are needed on the group-level parameters as well as the variance–covariance matrix. When translating source memory theory into MPT models, the priors we place on the multivariate normal distribution (i.e., means, variances, and covariances) deserve careful consideration. These parameters determine which MPT parameter values are deemed plausible both at the group level and the individual level. Thus, carefully chosen prior distributions should (1) be theoretically justified, (2) faithfully reflect expectations about group- and individual-level parameters in their original probability scale, and by extension (3) imply sensible predictions of group- and individual-level response rates.

Let us consider the Matzke–Klauer prior on the group-level means of the MPT parameters – a standard normal distribution. This prior is popular since it translates to a uniform distribution on the probability space implying that all values are equally likely a priori. However, since specific prior distributions can be derived from many psychological theories, the standard normal distribution is not ideal for many cases. For instance, if sources appeared equally often and were randomly assigned to items, source guessing *g* is most likely to be at or near chance level (i.e., .5) rather than strongly biased towards one source. The same applies to source memory *d* which is recollection based and difficult, thus not likely to be near 1 but also not likely to be at 0 for healthy adults.

**Fig. 2:**
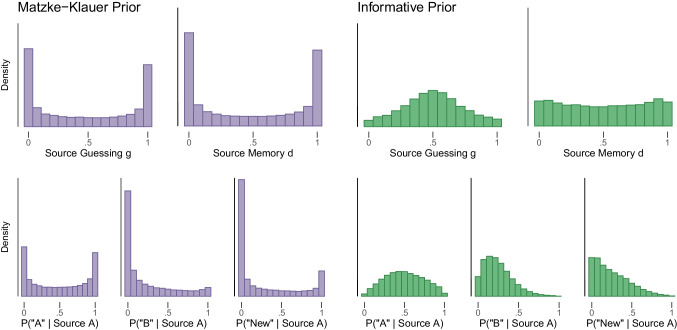
The 2HTSM model with Matzke–Klauer priors as implemented in TreeBUGS on the group-level leads to nonsensical and extreme predictions on the individual level (*left panel*; *purple*). The *right panel* shows (*in green*) predictions of the 2HTSM model with informative prior distributions. The top row shows for one participant prior predictions for the source guessing and source memory parameter. The bottom row depicts for one participant the prior predictions of the probabilities of responding that an item from source A stemmed from source A (*left*), source B (*middle*), or is a new item (*right*). Predictions were generated by drawing prior samples from the model

A less obvious problem is a vague prior on the variance–covariance matrix. It seems natural to assume that vague priors on the group-level means in combination with high participant variability will lead to vague priors at the individual level. Yet, when moving from the latent space to the probability space, these vague group-level priors combine to highly informative priors for individual participants.

The left panel in Fig. 2 illustrates the individual-level predictions of a participants’ source memory parameter and source guessing parameter as well as the predicted category probabilities to answer “A”, “B”, or “New” given that the correct source was Source A. These predictions indicate that the Matzke–Klauer priors place an outsized amount of prior probability mass on implausible extreme values, a perhaps unexpected result for many users of these models (Lee (2018) illustrated a similar case in the field of psychophysics). The prior distribution on individual-level *g* parameters posits that a participant is most likely to either always guess correctly or to never guess correctly. Similarly, the prior distribution on individual-level *d* parameters posits that a participant is most likely to either have perfect source memory or no source memory at all. Based on these priors, any plausible group-level parameter values may correspond to (symmetric or asymmetric) bimodal distributions of individual participant parameters. These priors clearly do not express theoretically sensible expectations about individual participants’ behavior.^2^

**Fig. 3:**
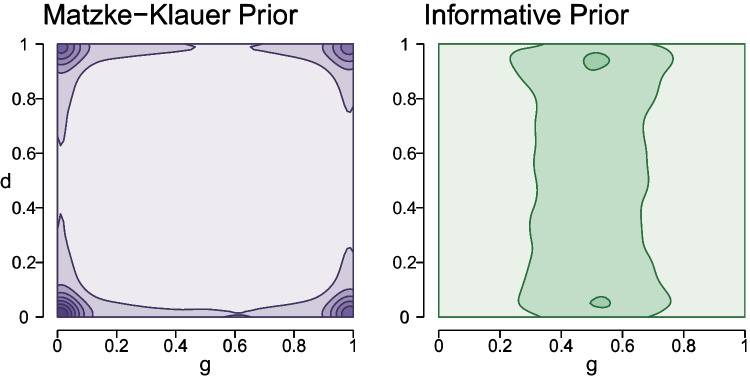
Illustration of the bivariate population distribution of participants for the source memory (*d*) and source guessing (*g*) parameters of the 2HTSM model with Matzke–Klauer priors as implemented in TreeBUGS (*left panel*; *purple*) and informative priors (*right panel*; *green*). *Darker colors* indicate a higher density. When assigning Matzke–Klauer priors, the model predicts a mixture of four populations. Each population realizes one extreme combination of guessing and source memory. In contrast, informative priors cover the space of possible values for *d* and *g* more evenly, that is, extreme values are favored less

Moreover, these priors are at odds with typical assumptions about the population distribution. A key motivation for using hierarchical models, such as the latent-trait model, is that they assume that participants belong to a relatively homogeneous population and that therefore the estimates of any one participant partially inform estimates of all other participants from the same population. In the original probability scale, the prior predictions of the Matzke–Klauer priors, however, implement an assumption that is antithetical to the assumption of a common population: a mixture of several different populations. The composition of this mixture is illustrated in Fig. 3. The figure shows the multivariate prior distribution for the individual-level *g* and *d* parameters of the 2HTSM model. The left panel shows the distribution for Matzke–Klauer priors. Here, the density is localized in the corners of the plot implying four populations of participants: (1) perfect source memory and never guessing “Old”; (2) perfect source memory and always guessing “Old”; (3) no source memory and never guessing “Old”; and (4) no source memory and always guessing “Old”. (In general, the model will predict a mixture of $2^k$ populations, where *k* is the number of parameters.) This pattern seems undesirable; after all, priors in line with MPT-modelers’ expectations would spread prior mass more evenly across all combinations of the two parameters instead of the extremes. Thus, the Matzke–Klauer priors neither yield sensible predictions for any single individual nor for the population distribution.

This problem becomes more serious the more vague the prior distributions are, which is why the original priors proposed by Klauer (2010) have been increasingly constrained over the years. While the original specification in Klauer (2010) allowed for the most participant variability, Matzke et al. (2015) proposed priors that were slightly more constrained. Heck et al. (2018) implemented in TreeBUGS the model proposed by Matzke et al. (2015), but constrained participant variability even further.

The predicted mixture distribution reduces hierarchical shrinkage of individual parameter estimates (i.e., partial pooling) relative to a more informed prior and can result in more extreme estimates. As a result, the peaked and extreme prior distributions requires more data to be overpowered. In fields such as memory research, the problem may be particularly serious as memory capacity is limited and therefore only a limited number of items can be presented to the participants (which is one reason why data in this field are often aggregated; Chechile (2009)). In such a scarce data environment, extreme priors may influence posterior estimates, especially the individual estimates.

### Refinement No. 1: Determine informative parameter priors

To prevent parameter priors from jeopardizing the modeling process, researchers need to pay due attention to their specification (Barnard et al., 2000; Lee & Vanpaemel, 2018). This concept can also be found in the TreeBUGS manual: Heck et al. (2018) encourage users to develop customize default priors, for instance, for the source guessing parameter *g*. Part of the model specification should be to place appropriate theory-based restrictions on the priors for group-level MPT parameters and on the variability between participants encoded in the covariance matrix. Importantly, our aim is not to advertise any particular alternative default priors. As Vanpaemel (2010, p. 495) states: “No formal guidelines about how to capture theory into a prior exist, just like there are no formal guidelines about how to capture theory into a model equation. Model building crucially depends on the skill and the creativity of the modeler and cannot be automated.” Instead of proposing default priors, we advise using visualizations of prior predictions at the group and individual level to guide the development of appropriate models (Gabry et al., 2019; Schad et al., 2021; Wagenmakers et al., 2021). An example for this practice in MPT research can be found, for instance, in Gronau et al. (2019).

Based on the above considerations, we have made the following adjustments to the Matzke–Klauer priors for the 2HTSM model. First, we replaced the uniform prior distribution for the guessing parameter with a prior distribution that mildly favors values around the nominal guessing level. Additionally, to counteract the extreme predictions about the population distribution, we placed stronger constraints on the participant variability encoded in the covariance matrix. To determine the exact prior distribution for the covariance matrix, we proceeded as follows. First, we explored a set of prior distributions on parameters describing individuals’ deviations around the group-level mean and their correlation, which adequately implemented our assumption that participants are relatively similar. This resulted in a total of 48 plausible prior settings. Then, we conducted a small simulation study in which we visualized the predictions for data from these priors. We settled on priors that yielded individual-level predictions that were uninformative and had little bimodality. The set of priors that resulted in the best model predictions is the one depicted on the right panel in Figs. 2 and 3. In this model, small deviations from the group-level means are favored over large ones, resulting in a more homogeneous population. In addition, our priors favor moderate-to-low correlations between MPT parameters in the range of $[-0.5, 0.5]$ over anything more extreme. The constraints aim to reduce but not completely eliminate the likelihood of extreme parameter values. By imposing these restrictions, the model now makes moderate predictions, which are better in line with our basic intuitions and theoretical expectations.

## Testing informative ordinal predictions

The previous section outlined the role of informative prior distributions for the general model specification. This section explains how to make use of informative prior distributions for model comparisons in the context of a particular study design. That is, we further adjusted parameter priors across experimental conditions so that they conform to informative hypotheses. In particular, we focus on hypotheses on specific orderings of parameters across conditions, that is, ordinal or disordinal interactions.

A disordinal interaction was predicted, for instance, in Bell et al.’s study on biases. Specifically, the authors investigated how appearance-based biases would affect person memory. The authors were interested in a directional model, which predicted that unexpected information should be remembered easier than expected information. Furthermore, the effect should differ across two experimental conditions. That is, the effect should be larger if the unexpected information was positive than if the unexpected information was negative. An example of ordinal interactions that extend over several experimental conditions can be found in the study by Symeonidou and Kuhlmann (2021). Symeonidou & Kuhlmann (2021) proposed a test which measures source recognition and thus is more sensitive to source storage manipulations than the commonly used test. The authors predicted therefore that their test would outperform the standard test. In addition, they predicted that this added benefit was influenced by how often subjects were presented with the source–item pair and by the types of sources used. Overall, then, predictions were made about the ordinal relations between one main effect and two interactions (i.e., the benefits of the novel test should be *larger* in one condition than another).

Frequentist approaches test ordinal and disordinal interactions through reparametrization of the MPT parameters (Knapp & Batchelder, 2004) but so far have only been applied for non-hierarchical models and interactions between no more than two factors (e.g., Kuhlmann et al., 2019; Moshagen, 2010; see Klauer et al., 2015 for an application to confidence or Likert scales). In principle, within the Bayesian framework disordinal interactions can also be implemented through reparametrization. However, this technique adds a layer of complexity to the assignment of appropriate prior distributions, since the reparameterized model requires adjusted priors that are coherent on the original scale (Heck & Wagenmakers, 2016). Moreover, so far the literature on Bayesian MPT models has focused primarily on parameter estimation methods but not on model comparison by means of the Bayes factor (Jeffreys, 1935; Kass & Raftery, 1995).

### Refinement No. 2: Specify ordinal expectations as competing statistical models

Determining the Bayes factor for cognitive models is computationally complicated. However, when expectations are expressed in the parameter priors directly and they concern either point null hypotheses or directional hypotheses (e.g., interactions), the problem can be greatly simplified. The methods discussed here have three important advantages over the frequentist method. First, there is no need to reparameterize the MPT parameters in order to represent interaction effects, which facilitates the interpretation of the estimates and the assignment of prior distributions. Second, the methods are suited for hierarchical models, thus taking into account participant heterogeneity and hierarchical shrinkage. Third, the methods are able to test theories directly. Interaction effects such as the ones predicted in Bell et al. (2015) and Symeonidou and Kuhlmann (2021) are typically tested in a traditional ANOVA approach. That is, seeking to reject the null hypothesis that there is no interaction, which carries no information about the validity of the model. Only when subsequently analyzing the contrasts between the conditions does it become evident whether or not the data adhere to the predicted pattern. To maximize efficiency and theoretical information, however, it is desirable to test the predicted pattern simultaneously. The model comparison method described here is able to do so.

For model comparisons between a point null and the encompassing model (i.e., a model that imposes no constraints on the parameters), the Bayes factor simplifies to the Savage–Dickey density ratio (Dickey & Lientz, 1970; Dickey, 1971). For model comparisons between a directional prediction and the encompassing model, the Bayes factor simplifies to the unconditional encompassing Bayes factor (Gelfand et al., 1992; Klugkist et al., 2005; Sedransk et al., 1985).

**Fig. 4:**
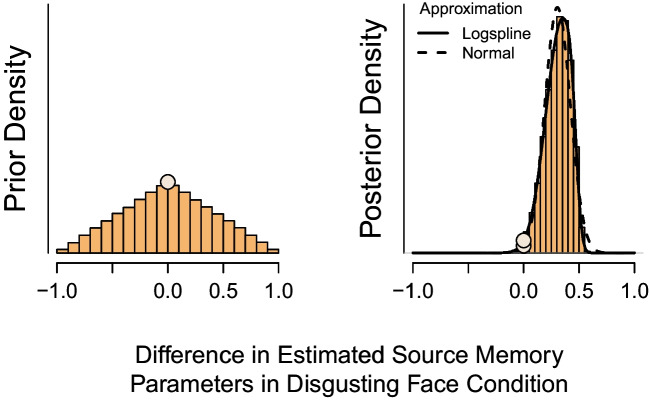
Estimates for the Savage–Dickey Bayes factor might vary depending on the method used to approximate the density. The histograms depict for the Bell et al. (2015) study, the prior and posterior density of the difference between source memory parameters in the pleasant face condition. For the prior distribution (*left panel*), the density is known. For the posterior distribution (*right panel*), the density needs to be approximated. The *solid line* represents the estimated logspline nonparametric density of the source memory difference, the *dashed line* the fitted truncated normal density of the source memory difference. The *dots* show for each approximation method the estimated height of the difference distribution at the zero point, that is, the point at which the source memory parameters are equal

#### Testing equality constraints

The Savage–Dickey Bayes factor is defined as the ratio of prior and posterior density under the encompassing model at the point of interest. To illustrate the approach, consider Bell et al.’s null model predicting that source memory is equal for unexpected and expected information in both experimental conditions. In this case, for the two experimental conditions, the point of interest is the zero point for the difference between the source memory parameters.

To test this prediction, for both experimental conditions the height of the prior and posterior density at zero needs to be computed. If the height of the density at zero is larger for the prior density than for the posterior density, it implies evidence in favor of the encompassing model. In this case, the data have caused more density to be allocated to a different part of the distribution. If the height of the density at the zero point is lower for the prior density than for the posterior density, this is evidence in favor of the null model. In this case, the data have caused values around the zero point to become more likely.

In some cases (mostly for prior densities) the height of the distribution at the point of interest is available in closed form and can thus be computed directly. To illustrate, consider the prior distribution of the difference between two group-level source memory parameters depicted in the left panel of Fig. 4. In Bell et al.’s study, each source memory parameter was assigned a standard normal distribution on the probit scale which translates to a uniform $\text {Beta}(1, 1)$ distribution on the probability scale. The distribution of the difference thus translates to a difference between two uniform beta distributions, which has a triangular shape with density 1 at the zero point.

In cases where the distributions cannot be obtained in closed form (mostly posterior densities) the density at the point of interest needs to be approximated using Markov chain Monte Carlo (MCMC) samples. The approximated density can then be calculated, for instance, using logspline nonparametric density estimates (Stone et al., 1997; Kooperberg, 2020). The logspline method has the advantage that it can approximate various forms of posterior distribution well, including bi-modal or highly skewed distributions. Since researchers can usually not predict the shape of the posterior distribution of their parameters before data collection, this is an advantage, especially if the aim is to receive the most accurate estimates. However, this close approximation to the posterior samples makes this method susceptible to sampling uncertainty and thus produces Bayes factors that are more variable.

As an alternative, modelers can opt to fitting a normal distribution to the MCMC samples using the methods of moments (Morey et al., 2011). To account for the inherent boundaries of difference parameters, the normal density can be truncated by setting lower and upper bounds at $-1$ and 1, respectively. The (truncated) normal approximation method generally yields more stable estimates since it requires only the mean and standard deviation of the posterior samples. This is a favorable property especially if the point of interest is located in the tails of the distribution. Both approximation methods are depicted on the right panel of Fig. 4. For our case study, we conduct the main analysis using the logspline approximation method but report, for completeness, also the results obtained using the truncated normal approximation method.

**Fig. 5:**
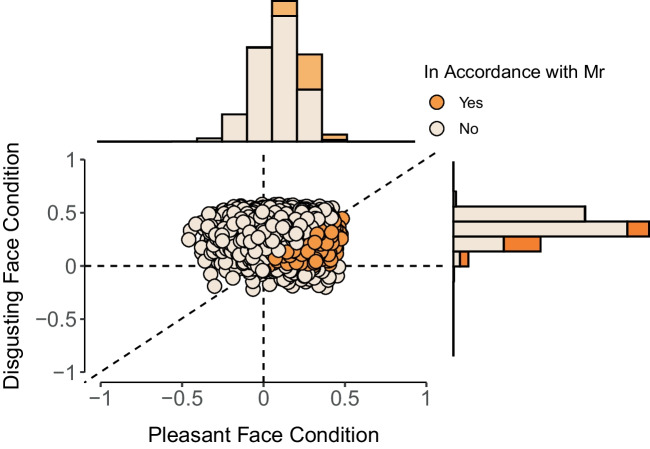
The unconditional encompassing approach estimates the Bayes factor through the proportion of prior and posterior counts in accordance with the restriction. The histograms represent for Bell et al.’s study the posterior source memory parameters in each condition separately, the scatterplot represents the joint distribution. *Orange color* reflects samples in agreement with the constrained model and *white color* represent samples that are not in agreement with the model

Note that computing the Savage–Dickey Bayes factor requires that the point null model can be derived from truncating the prior distribution of the encompassing model. Moreover, the Savage–Dickey Bayes factor, as described here, does not directly apply to testing the equality of more than two parameters simultaneously. However, it is feasible to test different parameters separately and make individual assessments for each one. Finally, the test-relevant parameters should be independent of all other parameters in the model (Wetzels et al., 2010; Heck, 2019). In MPT research, this requirement is met when (1) expectations concern group-level MPT parameters (e.g., group-level source memory under different experimental conditions) and (2) independent priors are assigned to these parameters, as is the case of the current instance of the 2HTSM model. Caution should be taken when testing parameters at a lower level, for instance, individual-level MPT parameters, as they are dependent of nuisance parameters (i.e., parameters that are unconstrained under the null model). In these cases, Bayes factors can be computed using the generalized Savage–Dickey density ratio (Verdinelli & Wasserman, 1995).^3^

#### Testing ordinal constraints

Let us turn to directional hypotheses. The Bayes factor identity we use to test ordinal constraints is a generalization of the Savage–Dickey density ratio (Wetzels et al., 2010). In contrast to the Savage–Dickey density ration, however, the unconditional encompassing method does not require an approximation of the prior or posterior densities. Instead, the unconditional encompassing method is a simple counting method. That is, the unconditional encompassing Bayes factor is defined as the ratio of the sample proportions of the prior and posterior draws of the encompassing model that match the restriction. Thus, only the number of prior and posterior MCMC samples obtained from the encompassing model are counted which satisfy the constraint.

Returning again to Experiment 2 in Bell et al.’s study, the directional model states that unexpected information should be remembered easier than expected information and that this effect should be larger in the pleasant face condition. When we have MCMC samples available across all conditions, we can evaluate whether the prediction is true for each iteration. The dots in Fig. 5 depict samples from the posterior distribution of the encompassing model for the difference of source memory parameters in both experimental conditions. The orange dots depict samples for which the prediction holds, that is, the difference in the source memory parameter for unexpected information and expected information is positive and that the difference between the source memory parameters is larger in one experimental condition than the other.

The proportion of prior draws in accordance with the restricted model can be computed directly. Since the distribution of the difference between source memory parameters is symmetric at zero, in each condition, half of the samples will be positive, that is, the source memory parameter for unexpected information and expected information is positive, which yields a proportion of $0.5^2 = 0.25$. From this subset, again, half of the samples from the pleasant face condition will be larger than for the disgusting face condition (i.e., $0.25/2 = 0.125$). Thus only the sample proportion of posterior draws in accordance with the restricted model needs to be computed.

Here again, if the proportion of prior samples in agreement with the constraint is smaller than the proportion of posterior samples in agreement with the constraint, it implies evidence in favor of the restricted model. Vice versa, if the proportion of prior samples in agreement with the constraint is larger than the proportion of posterior samples in agreement with the constraint, it implies evidence against the restricted model. What makes this method user-friendly is that this simple counting principle can be generalized to highly complex directional hypotheses involving multiple conditions.

#### Scale dependence in ordinal constrained inference

A caveat to using reparametrization of the MPT parameters (Knapp & Batchelder, 2004) to test ordinal constraints is that results may change depending on whether parameters are tested in the probability or the probit space. That is, due to the nonlinearity of the probit transformation, (dis)ordinal effects may become more pronounced or disappear when transitioning between scales (so-called removable interactions), which may lead to different conclusions.

As pointed out in Wagenmakers et al. (2010), the probability space changes at the boundaries of the scale (e.g., moving from 0.85 to 0.95) represent larger absolute changes compared to changes at the center of the scale (moving from 0.55 to 0.65; cf. logistic/probit regression). Testing hypotheses on the probit-space therefore has the advantage that it puts a weighting function on changes on the boundaries, which makes changes comparable. At the same time, probit-transformed MPT parameters are less interpretable than those on the probability scale and are often considered a mere technical convenience to model correlations and to incorporate covariates rather than being considered quantities to reason about. The interpretation of the parameters and the formulation of hypotheses often happens on the probability scale. Since Bell et al. (2015) directly related source memory (instead of probit transformed source memory) to behavior descriptions and facial appearance of the stimuli, we therefore decided to test the model parameters in our case study on the same scale.^4^

In addition to the interpretation of the parameters, the interpretation of the moments of source parameters are also scale-dependent. Since the probit to probability transformation is non-linear, the transformed group-level parameters $\Phi (\mu )$ cannot be interpreted as group-level means on the probability scale, but merely as group-level medians (see Heck et al. 2018). Additionally, the covariances between parameters on the probit scale do not directly translate to the covariances on the probability scale. In TreeBUGS, users have access to the probitInverse() function, which facilitates exploration of the relationship between parameters on the probit scale and their corresponding values on the probability scale.

## Case study

In this section, we illustrate parameter estimation and model comparison using informative priors for the study by Bell et al. (2015) who embedded source memory in a theory on schema-congruence.

### Model

The case study implemented a slightly adapted version of the 2HTSM model. In contrast to the model presented in Fig. 1, Bell et al. (2015) assumed that the item memory *D* and the source memory *d* for sources A and B are not necessarily equivalently good. Thus, their model estimated these parameters separately for the respective source. In addition, the probability of distractor detection of new items was set to the average item memory of source A and source B, that is, $D_{\text {New}} = (D_{\text {A}} + D_{\text {B}})/2$. Finally, the authors estimated separate MPT parameters in each of the two within-subjects conditions. Thus, for each participant, 12 MPT parameters were estimated, that is, two pairs of item memory and source memory parameters and two guessing parameters in each condition.

We implemented the hierarchical 2HTSM model with the modifications outlined in the previous sections and detailed in the appendix. That is, we assumed item homogeneity, assigned a $\text {Normal}(0, 0.28)$ prior distribution to the probit-transformed group-level guessing parameters, a $\text {Normal}(0, 1)$ prior distribution to the probit-transformed group-level memory parameters, a $\text {LKJ}(1)$ prior distribution to the correlation matrix, and a $\text {Gamma}(2,3)$ prior distribution to the standard deviation of the individual shift parameters.

**Fig. 6:**
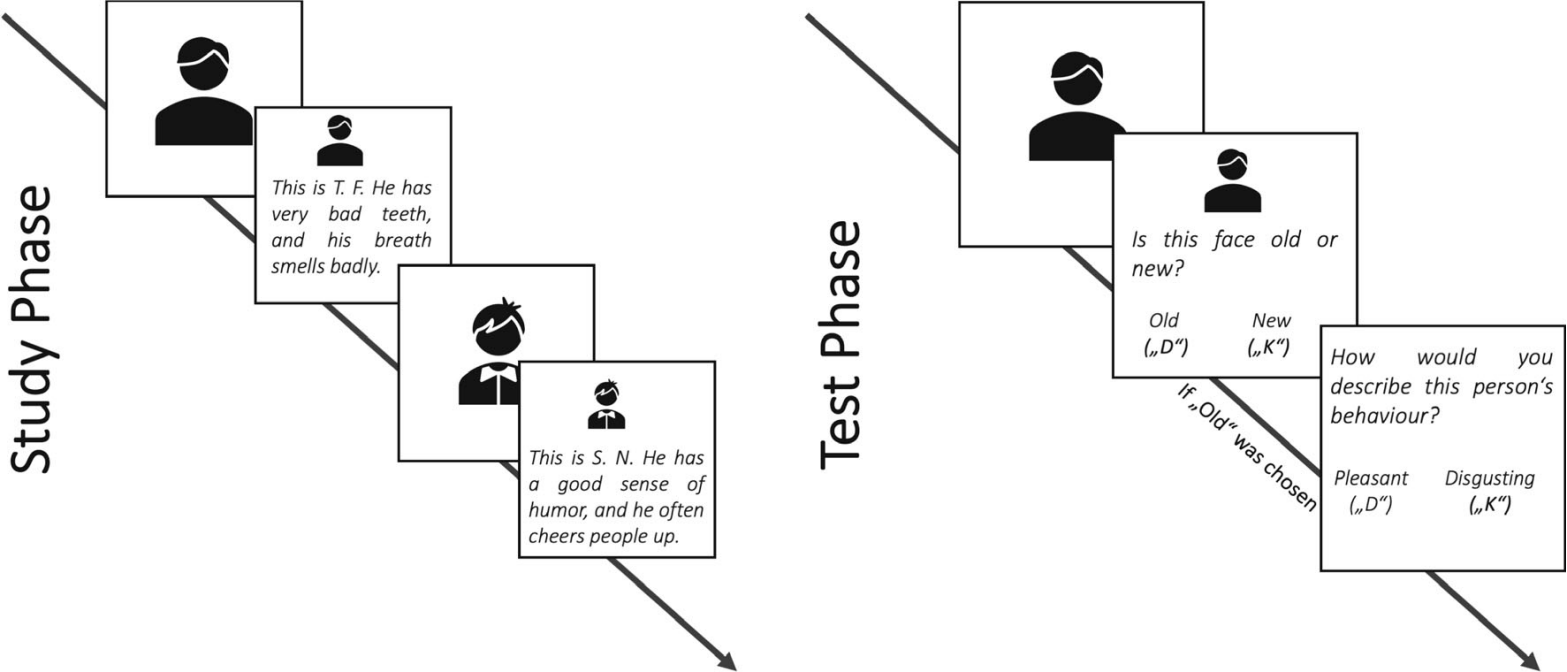
Schematic illustration of the experimental procedure in Bell et al. (2015) relevant to the reanalysis. In the study phase (*left*), participants were shown faces and behavior descriptions of persons that were either disgusting or pleasant. In the test phase (*right*), participants were presented with faces again. If participants indicated that they had already seen the faces in the study phase, they had to indicate whether the person’s behavior was disgusting or pleasant

### Method

Parameter estimation and model comparison was based on 50,000 samples from the posterior distribution of the encompassing model with 10,000 samples as burn-in. In both case studies, the relevant comparison was between the null model and the restricted model expressing ordinal predictions. In addition to the main analysis, we report the range of Bayes factors that resulted from a sensitivity analysis in which we assigned any other of our 48 prior specifications. We also report the Bayes factor between the encompassing model and the restricted model as a “bookend” comparison. Bookends in model comparison have been suggested as a proxy of model adequacy; that is, a model is only considered adequate if it can outperform models that are more parsimonious (e.g., the null model) and models that are more complicated (e.g., the saturated model which fits the data perfectly if the goal is to assess general adequacy of the model; Lee et al. (2019)). Here we aimed to ensure that, within the proposed model, the effects of the manipulation point in the expected direction in the sense that they are within the model comparisons we are running. Therefore, we select the encompassing model as a bookend, instead of a saturated model. Finally, we report normal approximation results to illustrate method uncertainty.

We first computed the Bayes factor of the null versus the encompassing method using the Savage–Dickey density ratio. We then computed the Bayes factor of the restricted versus the encompassing model using the unconditional encompassing Bayes factor. With the two quantities at hand, the desired comparison between the null and the restricted hypothesis was obtained through transitivity. For the main hypothesis (i.e., the hypothesis concerning group-level parameters) and informative priors we computed the Bayes factor 100 times to quantify the computational stability of the Bayes factor estimates (i.e., repeatedly sampled from the posterior distribution). For Matzke-Klauer priors Bayes factors were computed 20 times. We report the median of the Bayes factor estimates and their range. Bayes factors used to illustrate a concept (i.e., Bayes factors assessing individual differences, sensitivity analyses) were computed only once.

### Reanalysis of Bell et al. (2015)

To illustrate our approach for the case of disordinal interactions, we conducted a reanalysis of the data from Experiments 1 and 2 presented in Bell et al. (2015). Both experiments investigated how appearance-based first impressions affect person memory. Figure 6 shows a schematic illustration of the experimental setup. In the study phase, participants were instructed to memorize person information, that is, pictures of faces and short behavior descriptions. Importantly, the faces were chosen to be either pleasant-looking or disgusting-looking. Similarly, the behavior descriptions were categorized as being either pleasant or disgusting. Thus, within these combinations, the behavior description either matched the appearance of the face (congruent condition) or not did not match the appearance (incongruent condition). In the subsequent test phase, participants were presented with pictures of faces again. Their task was to indicate whether they had seen the faces before and if so, whether their behavior had been pleasant or disgusting.

#### Data

The individual-level data for the study were kindly provided by the authors. The experiments feature data from 138 and 114 participants, respectively. Each participant was instructed to learn 40 face–behavior pairs randomly drawn from an item pool, with 10 falling into each of the four cells of the experimental design. In the test phase, 40 additional faces were introduced. Thus, each participant provided a total of 80 data points.

**Fig. 7:**
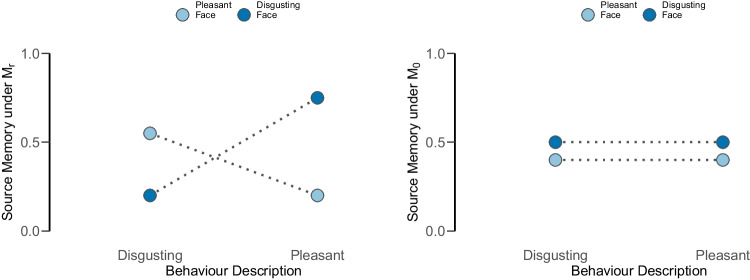
Schematic representation of the group-level source memory parameter under the restricted model (*left*; $\mathcal {M}_r$) and the null model (*right*; $\mathcal {M}_0$). The restricted model makes predictions both about the ordering of the source memory parameters within the behavior description conditions (*x*-axis) but also in within the faces conditions (depicted as *bright* and *dark blue dots*). The null hypothesis predicts that the source memory should be equal for both behavior descriptions

#### Hypothesis

The authors hypothesized that unexpected information should be memorized easier than expected information (see Fig. 7). This hypothesis was based on the findings by Bell et al. (2012) who suggested the existence of a cognitive mechanism which emphasizes events that contradict expectations. Moreover, the authors found that the effect was larger when positive expectations were violated. The authors attributed this interaction to the fact that subjects likely expected more negative behavior descriptions and that therefore unexpected positive behavior descriptions made a stronger impression on the participants.^5^ We compared the restricted model that describes this disordinal interaction to a null model that predicts that inconsistent and consistent information is remembered equally well for both face conditions.

#### Model fit

**Fig. 8:**
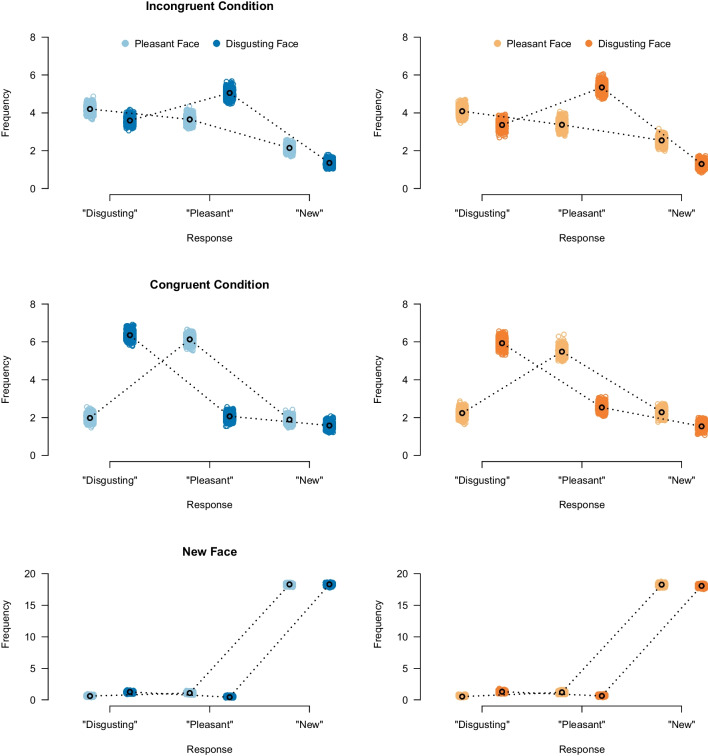
Posterior model predictions of data in Experiment 1 (*left*) and Experiment 2 (*right*). In the learning phase, participants learned face–behavior combinations. Within these combinations, the behavior description either not matched the appearance of the face (incongruent), or matched their appearance (congruent). In the test phase, participants were shown faces again, together with new faces. Each panel shows the participants’ observed and predicted responses during the test phase. The frequencies indicate the number of participants responding with "disgusting behavior description", "pleasant behavior description", or whether they indicated that the presented face was new. The *colored dots* reflect the models’ predicted responses. The *black dots* represent the observed response frequencies. The model using informative priors closely matches the observed responses indicating good model fit

Before reporting the Bayes factors, we first examine model fit indices to ensure that our model can adequately describe the data. Figure 8 displays posterior model predictions from our model in both experiments. The posterior predictive distribution can be interpreted as the model’s attempt to re-describe the data that yielded the posterior distribution. An adequate model should be able to reproduce the data used to update its prior distributions; the figure suggests that the model predictions perfectly match the observed response frequencies. This conclusion is confirmed by quantitative measures of model fit such as the T1 and T2 statistics proposed by Klauer (2010). That is, the model is able to recover the observed mean structure ($p_{T1} = 0.397$ for Experiment 1 and $p_{T1} = 0.469$ for Experiment 2) as well as the observed covariance structure ($p_{T2} = 0.261$ for experiment 1 and $p_{T2} = 0.361$ for Experiment 2).^6^

#### Results

**Fig. 9:**
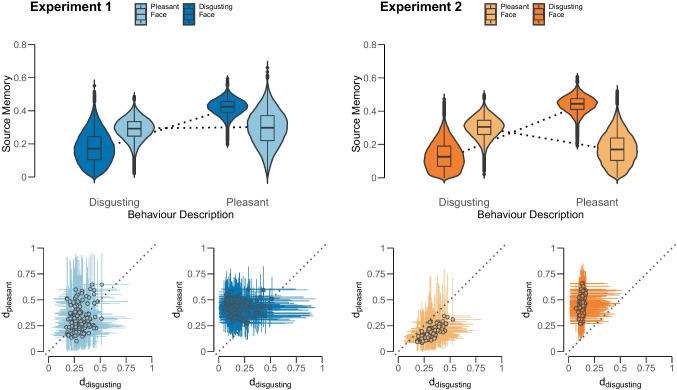
Violin plots of the estimated source memory parameters in Experiment 1 (*left panel*; *blue*) and Experiment 2 (*right panel*; *orange*) of Bell et al. (2015). In the *top panels*, we illustrate the group-level parameters. The *bottom panel* illustrates the comparison between the source memory parameters in the two behavior description conditions at the individual level. The *dots* represent the median estimate and the *error bars* the 80% credible intervals

The group-level parameter estimates and the individual-level parameter estimates are displayed in Fig. 9. The Bayes factors are summarized in Table 1. For Experiment 1, the data are uninformative. That is, the Bayes factor in favor for the null model relative to the restricted model centers around $\text {BF}_{r0} = 0.51$ and ranges from 0.45 to 0.57. This result is robust against alternative prior specifications and methods used to test equality constraints. For Matzke–Klauer priors, the evidence is equally weak but points in the opposite direction, with a Bayes factor in favor of the restricted model which centers at $\text {BF}_{r0} = 2.06$, ranging from 1.65 to 3.14. In the bookend comparison between the restricted and the encompassing model, the data too are uninformative, suggesting that the restricted model cannot outperform the encompassing model. To conclude, the Bayesian analysis does not support (but also does not contradict) the results from the frequentist analysis of the aggregate data reported by Bell et al. (2015) which suggested the presence of a disordinal interaction. This discrepancy may be partially explained by the fact that the parameter estimates from the hierarchical analysis are very uncertain, as can be seen in the left panel in Fig. 9. Although the point estimates descriptively conform to the predicted disordinal interaction, the large uncertainty in the estimates leads to inconclusive evidence.

**Table 1:** Summary of computed Bayes factors for Bell et al. (2015). The null hypothesis was evaluated using both the logspline approximation method and the truncated normal approximation method to compute the Savage–Dickey Bayes factor $\text {BF}_{r0}$

	BF type	Experiment 1		Experiment 2	
Group-level					
Main analysis (informative)	$\text {BF}_{r0}$	0.51	[0.45, 0.57]	4.4	[3.77, 5.71]
Main analysis (Matzke–Klauer)	$\text {BF}_{r0}$	2.06	[1.65, 3.14]	8.16	[5.75, 11.59]
Sensitivity analysis	$\text {BF}_{r0}$	–	[0.42, 1.29]	–	[3.42, 7.17]
Bookend comparison	$\text {BF}_{re}$	0.71	[0.64, 0.77]	1.19	[1.12, 1.28]
Individual-level comparison					
informative	$\text {BF}_{re}$	0.0011	–	0.45	–
Matzke–Klauer	$\text {BF}_{re}$	43.34	–	3,444	–
Group-level (Normal-Approximation)					
Main analysis (informative)	$\text {BF}_{r0}$	0.55	[0.49, 0.63]	6.99	[5.98, 8.55]
Main analysis (Matzke–Klauer)	$\text {BF}_{r0}$	2.87	[1.94, 6.51]	44.47	[27.09, 61.69]
Sensitivity Analysis	$\text {BF}_{r0}$	–	[0.45, 1.77]	–	[6.14, 31.87]

*Note.* For each test (rows) and experiment (columns) the Bayes factor in favor of the restricted model versus the null ($\text {BF}_{r0}$) or the encompassing model ($\text {BF}_{re}$). Numbers in square brackets indicate Bayes factor ranges. See the main text for an explanation of the different tests. Empty cells indicate that the quantity was not computed for a particular analysis

**Table 2:** Estimates for the group-level source memory parameter *d* for the data of experiment 1 and 2 in Bell et al. (2015). The column “Reported” shows the estimates as reported in the original manuscript using frequentist estimation on aggregated data. The columns “informative” and “Matzke–Klauer” show the median estimates and 95% credible intervals when using Bayesian hierarchical approaches

			Source memory *d*		
	Face	Behavior	Reported	Informative	Matzke–Klauer
Experiment 1	Pleasant	Pleasant	.35 [.20, .50]	.30 [.08, .49]	.20 [.01, .43]
		Disgusting	.31 [.21, .41]	.29 [.15, .40]	.32 [.18, .42]
	Disgusting	Pleasant	.49 [.43, .55]	.42 [.32, .51]	.42 [.30, .50]
		Disgusting	.03 [.00, .30]	.17 [.02, .37]	.11 [.01, .33]
Experiment 2	Pleasant	Pleasant	.05 [.00, .30]	.17 [.02, .36]	.17 [.02, .37]
		Disgusting	.40 [.31, .49]	.31 [.16, .41]	.27 [.07, .39]
	Disgusting	Pleasant	.47 [.40, .55]	.44 [.33, .53]	.45 [.35, .53]
		Disgusting	.10 [.00, .32]	.13 [.01, .31]	.08 [.00, .27]

*Note.* Data of reported estimates are extracted from Figs. 3 and 4 of the original manuscript

**Table 3:** Estimates for the group-level source guessing parameter *g* for the data of experiment 1 and 2 in Bell et al. (2015). The column “Reported” shows the estimates as reported in the original manuscript using frequentist estimation on aggregated data. The columns “informative” and “Matzke–Klauer” show the median estimates and 95% credible intervals when using Bayesian hierarchical approaches

		Source guessing *g*		
	Face	Reported	Informative	Matzke–Klauer
Experiment 1	Pleasant	.36 [.30, .42]	.35 [.27, .43]	.32 [.24, .41]
	Disgusting	.75 [.69, .80]	.69 [.62, .76]	.69 [.61, .75]
Experiment 2	Pleasant	.30 [.24, .37]	.36 [.29, .43]	.37 [.30, .45]
	Disgusting	.67 [.61, .73]	.66 [.59, .72]	.68 [.61, .73]

*Note.* Data of reported estimates are extracted from Table 4 in the original manuscript

For Experiment 2, the data suggest moderate evidence in favor of the restricted model relative to the null model. The Bayes factor centers around 4.4 and ranges from 3.77 to 5.71 and is robust against different prior specifications. These estimates are smaller and less variable compared to the ones we receive with the Matzke–Klauer prior, for which Bayes factor estimate centers at 8.16, ranging from 5.75 to 11.59, which suggests moderate-to-strong evidence in favor for the restricted model. As for the bookend model, the data again are uninformative suggesting that the restricted model is not able to outperform the encompassing model. Thus, for the second experiment, the Bayesian analysis supports the results from the frequentist analysis and suggested the presence of a disordinal interaction.

Notably, not the choice of prior distributions but the method used to compute the Savage–Dickey Bayes factor has the biggest effects on the results; for the normal approximation method Bayes factors go as high as 62 in favor for the restricted model using the Matzke–Klauer prior. When assessing the robustness of the Bayes factor under different prior specifications for informative priors, values go as high as 32 in favor for the restricted model. These findings suggest very strong evidence for the presence of the disordinal interaction. These large differences may stem from the skewed distribution of the source memory parameter in the ’disgusting-face, disgusting-behavior’ condition (visible in the violin plot in Fig. 9) so that the difference in source memory parameters in the disgusting face condition cannot be accurately described using only the mean and standard deviation. This in combination with the Matzke–Klauer prior positioning the parameter closer to the boundaries ($d = 0.08$) than the informative prior ($d = 0.13$), accentuates the interaction even further.

#### Parameter estimates

Table 2 summarizes the estimates for the source memory parameter, obtained from informative prior distributions, the reported estimates in the original manuscript, and from Matzke–Klauer priors. The reported estimates – which were based on frequentist estimation on aggregated data – suggest less variability in the estimates compared to the Bayesian hierarchical models. The two Bayesian models are more similar to each other with the exception of the source memory estimates regarding the pairing of disgusting face and disgusting behavior in both experiments. Although all estimates have similar credible/confidence intervals with lower bounds close to zero, the median of the informative prior is higher than the estimates from the other approaches. Regarding the source-guessing parameter (Table 3), the estimates mostly converge. Experiment 1 shows a slight discrepancy in the parameters in the disgusting face condition. Here the two Bayesian models yield similar estimates that are smaller than the reported ones.

#### Assessing individual differences

Another benefit of Bayesian hierarchical models is that they estimate overall effects, but are also suited to assess individual differences. When predicting a specific pattern of effects on cognitive parameters, researchers might be interested in whether the patterns observed on the aggregate level also generalize to the individual level (Haaf & Rouder, 2019; Miller & Schwarz, 2018). That is, whether biases on the population-level, are exhibited by all individuals within the population. Note that the studies by Bell et al. (2015) were designed to test group-level hypotheses, that is, the authors did not pose research questions concerning individual-level effects. As will become apparent below, the individual-level parameter estimates are also highly uncertain, which hampers statistical inference. Nevertheless, researchers interested in assessing individual-level effects in paradigms where more trials can be assessed might find the following demonstration valuable.

To study individual effects in the within-subject conditions (i.e., the simple effect of congruency within each experimental condition), we can assess whether descriptively the point estimates depicted at the bottom panel in Fig. 9 crossed the diagonal line contrary to the prediction. For Experiment 1, based on the median point estimates, $50.7 \%$ of participants (i.e., 70/138) showed the predicted effect for pleasant faces, that is, they remembered the pleasant face better when it was paired with disgusting (i.e., unexpected) rather than with pleasant information. For disgusting faces, this was the case for $92.80 \%$ of participants (i.e., 128/138). However, when accounting for the uncertainty of the estimates (i.e., 80% credible intervals), this number dropped to $0 \%$ in the pleasant face condition and $15.9 \%$ of participants (i.e., 22/138) in the disgusting face condition.

For Experiment 2, we see a similar pattern. For pleasant and disgusting faces, all participants (i.e., 114/114) showed the predicted effect, but when accounting for the $80\%$ credible intervals, this number dropped to $0 \%$ (i.e., 0/114) in the pleasant face condition and to $48.2 \%$ (i.e., 55/114) in the disgusting face condition.

#### A principled test to evaluate individual differences

Although the descriptive statistics give an insight into whether or not the individuals show the predicted effect, assessing whether credible intervals cross the diagonal is not a principled test of whether all participants show the predicted pattern. Moreover, it yields only limited information about whether people outside of this sample would have an effect in the same direction. For a detailed discussion of the issues with this approach, we refer interested readers to Haaf and Rouder (2019); Thiele et al. (2017). Instead of counting the number of participants who show an effect in one or the other direction, we apply the model comparison approach developed by Haaf and Rouder (2017), and compare an individual-constrained model where *every* participant shows an effect in the predicted direction with an encompassing model where this constraint is not obeyed. If the encompassing model is supported, further research could shed light on the conditions under which the test is beneficial or not (Haaf & Rouder, 2017).

To examine individual differences, the same restrictions that we have previously imposed at the group-level can be applied at the individual level. That is, we now test whether the predicted biases in person memory are present in every participant at the same time. This model makes a very risky prediction: the a priori probability that all participants show the effect is only approximately five in 10,000 in both experiments, and in fact, the risk-taking does not pay off. Since the posterior probability that all participants show the effect is smaller than the prior probability (i.e., one in a million for Experiment 1 and three in 10,000 for Experiment 2) the hypothesis that all participants show the effect simultaneously is not supported. For Experiment 1, the data suggest extreme evidence in favor for the encompassing model with a Bayes factor of 942. For Experiment 2, the data suggested anecdotal evidence: the Bayes factor in favor for the encompassing model relative to the restricted model is 2.21.

#### Sensitivity of individual-level Bayes factor to group-level priors

So far, in this case study the differences in informative priors compared to Matzke–Klauer priors led to only negligible deviations in group-level estimates and the conclusions that one would draw from the Bayes factors of both priors differed only when method uncertainty was taken into consideration. However, the influence of the two priors becomes apparent when evaluating the individual effects.

For Experiment 1, with informative priors, the data suggest extreme evidence with a Bayes factor of $1/0.001 \approx 910$ *against* the restrictive model. By comparison, the Matzke–Klauer lead to the opposite conclusion, suggesting very strong evidence *in favor of* the restricted model relative to the encompassing model with a Bayes factor of 43. For Experiment 2, while the data suggest anecdotal evidence with a Bayes factor of $1/0.45 \approx 2.21$ *against* the restrictive model with informative priors, the Matzke–Klauer Bayes factor *in favor of* the restricted model relative to the encompassing model was 3444, suggesting extreme evidence.

These vastly different Bayes factors are a result of extreme participant populations predicted by Matzke–Klauer priors. The mixture of several populations causes an extreme low a priori probability that all participants will show the predicted effect simultaneously, approximately only one in 10 million for Experiment 1 and 2 (which is 1000 times less than for informative priors). The implication of this low a priori probability is that a small number of posterior samples consistent with the constraint is already sufficient to suggest evidence in favor of the restricted model, and indeed, since the posterior probability increased relative prior probability (i.e., 22 and 1768 in 10 million), the restricted model is favored in both experiments.

**Fig. 10:**
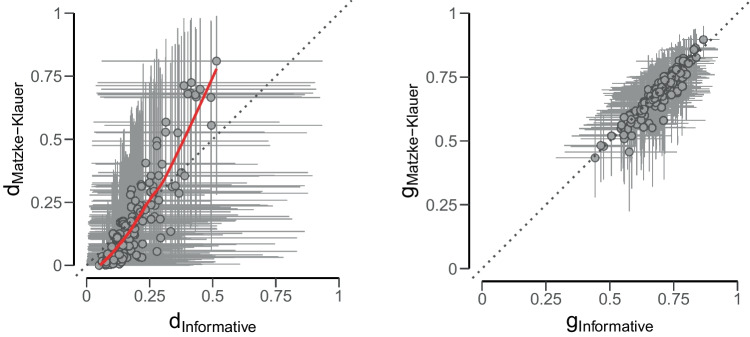
For the reanalysis of Bell et al. (2015), Matzke–Klauer priors as implemented in TreeBUGS and informative priors yield the same posterior estimates for guessing parameters (*right*). For the source memory parameters (*left*), on the other hand, the variance of the estimates from Matzke–Klauer priors is larger, which implies a smaller degree of shrinkage

When estimating individual-level parameters, the two priors also diverge. Figure 10 compares the individual estimates of source memory and guessing from the two models in the disgusting face-disgusting behavior condition in Experiment 1. The dashed line corresponds to identical estimates from the two priors. The estimates for source guessing parameters (right plot) do not deviate much from this line. For the source memory parameter (left plot), on the other hand, parameters estimated with Matzke-Klauer are closer to the extremes, while parameters estimated with informative priors are placed closer to the group mean. To reach a definite conclusion, the estimates obtained from the two priors could be compared to ones obtained maximum-likelihood estimates. Due to the scare data structure, however, the frequentist methods fail to estimate individual-level parameters.

## Discussion

This article discussed two points of refinement for the specification and comparison of Bayesian MPT models. We highlighted how to specify informative predictions both in terms of plausible values for model parameters within experimental conditions and in terms of the rank ordering across experimental conditions. We did do so by using the 2HTSM model for source monitoring, however, our arguments and methods generalize to all MPT research. The aim of this work was to provide researchers with principles for specifying and comparing MPT models and to present methods that are simple to use.

### Effects of prior selection on Bayes factors

To ensure plausible model predictions within experimental conditions, we argued for the need of informative priors. Using prior-predictive checks, we illustrated that the priors proposed by Matzke et al. (2015) and Klauer (2010) and implemented in the R package TreeBUGS (Heck et al., 2018) are in our application uninformative and vague at the group level but are highly informative at the individual level and favor extreme values. These distributions predicted nonsensical response rates and – instead of a homogeneous population of participants – a mix of different participant populations. Moreover, the priors affected Bayes factor estimates; in our case study, Matzke–Klauer Bayes factors and Bayes factors obtained from informative priors diverged, sometimes with extreme evidence for opposite hypotheses when the hypothesis concerned individual differences.

We presented various techniques to compute Bayes factors, however, Bayes factors are often criticized as they are sensitive to priors (e.g., Kass and Raftery, 1995). This is correct; as our case studies demonstrated, priors drastically affected the Bayes factors. Yet, the same applies to the other two steps of model specification. A different model equation will result in different Bayes factors, as will different hypotheses as they are incorporated in the model (see e.g., van den Bergh et al. 2022). In the formalization of theories, subjectivity comes into play at all stages of model specification – this is one of the characteristics of cognitive modeling and the construction of psychological theories in general. However, one should not equate subjectivity with randomness. The model equation, model assumptions, and parameter priors are not random: they result largely from theoretical considerations.

From our own experiences, MPT modelers are often discouraged from informing their priors for fear of being accused of “Bayes factor hacking”. Researchers can counteract this by justifying their parameter priors and preregister them to ensure the confirmatory status of their analyses. For aspects of the prior that are not fully justified by theory, researchers can perform sensitivity analyses to determine the extent to which their results are fragile or robust with respect to different modeling choices (Myung & Pitt, 1997; Sinharay & Stern, 2002). For instance, in our implementation of the 2HTSM model, our goal was to constrain the individual variability encoded in the covariance matrix; however, we considered several combinations of parameter values to be plausible. In this case, a sensitivity analysis could reveal whether the Bayes factor was robust against alternative plausible prior distributions; that is, whether different priors lead to diverging conclusions. When conducting sensitivity analyses, the alternative prior distributions should have the same theoretically justified properties as the prior distribution chosen in the analysis (i.e., a distribution centered around chance), make reasonable prior predictions, Haaf and Rouder (2017, 2019); Lee & Vanpaemel, 2018), and be preregistered along with the prior distribution chosen in the analysis. Sensitivity analyses are particularly justified when data are scarce (e.g., in memory research or research on clinical populations) and parameter priors can be expected to have a greater impact on the results.

### Effects of prior selection on parameter estimation

Furthermore, our case study showed some differences in the parameter estimates for the two priors. However, differences in parameter estimation did not affect all parameters to the same extent, instead they became apparent only in individual-level parameters. This is good news for MPT modelers: regarding parameter estimation on the group level, the two priors discussed in this article largely converged to the same posterior estimates. At the individual level, differences between the two priors were most pronounced for parameters that were subject to greater uncertainty.

In MPT models, the tree architecture gives an indication on which parameters might be affected most: the more frequently parameters occur in the individual branches, the more information is available for estimation. For instance, Fig. 1 shows that in the 2HTSM model, source guessing is featured in every branch as guessing can guide all responses. Thus, data from all trials can be used to estimate the parameter resulting in precise estimates. By contrast, source memory may be represented only in branches of previously presented items for which the source was correctly identified. MPT modelers should keep this in mind, especially when working with tree structures where the test-relevant parameters are the ones occurring in only a few branches. To inform these parameters, researchers could collect more data. However, in memory experiments, this is inherently difficult, since presenting participants with hundreds of source–item pairs for learning and retrieval is not possible. Alternatively, researchers have the option to enhance the informativeness of their MPT models by implementing design optimizations, as suggested by Heck and Erdfelder (2019). This involves carefully selecting items that guarantee an adequate number of responses within the relevant tree branches. Generally, in paradigms with scarce data and/or a tree-architecture that does not sufficiently inform test-relevant parameters, the development of informative prior distributions is especially important.

### Prior selection is research design-dependent

Here, we are not arguing against the use of the software package TreeBUGS. On the contrary, we welcome that its user-friendliness facilitates the wide application of Bayesian MPT models. In fact, in TreeBUGS Heck et al. (2018) have already further restricted the vague priors proposed by Klauer (2010) and Matzke et al. (2015). That TreeBUGS priors were not suitable for our application does not necessarily mean that this will be the case for other applications and MPT models.

Importantly, we do not propose our priors as a new default. The appropriateness of the priors depends on the specific MPT model and research design at hand. The specific prior we set on the correlation matrix, for instance, will make different predictions when more or fewer parameters are featured in the model. Furthermore, the priors chosen here, while making assumptions consistent with our theoretical expectations (e.g., source guessing centered at nominal guessing level), are far from perfect. As apparent in Fig. 2 our priors on the source memory parameters are not completely flat but still assign a higher density to extreme values. This is due to our consideration to adapt the default priors only to the extent that the model made reasonable prior predictions. However, based on the prior predictions, it would also be legitimate to constrain the model priors even further. That is, constraining the participant variability group level will further lead to predictions that result in a more homogeneous population.

Experienced MPT modelers will certainly be able to identify further improvements based on published literature or their own research. For instance, the model employed in this study assumes independent priors on the model parameters. While this approach is simple and common, using correlated prior distributions on group-level parameters is a more direct and flexible approach when testing hypotheses about condition differences. By placing a prior on a difference parameter, researchers can express expectations about condition differences independently of the average performance. This parameterization leads to correlated predictions about condition means: for instance, if source memory is high in the one condition, it should also be relatively high in the other condition, irrespective of the magnitude of condition differences. For such parameterizations, it is useful to distinguish between test-relevant and nuisance parameters and possibly use less informed priors for the latter. Gronau et al. (2021) demonstrated the substantial impact of considering parameter dependencies on the resulting Bayes factor. Future research could explore adjustments to our model to accommodate this possibility.

Furthermore, our model specification of MPT parameter correlations within subjects across conditions could certainly be refined. We placed an LKJ prior on the correlation matrix MPT parameters across within-subject conditions. Greater flexibility could be achieved through the construction of a more informative prior on the correlation matrix. For instance, this could involve assigning a uniform LKJ prior on the correlation matrix and normal distributions on its elements, as suggested by Martin (2021). This would allow researchers to directly model expectations about correlations between certain parameter pairs. For instance, item memory may be highly correlated within a subject across conditions while source memory might be affected by experimental manipulations.

Further improvements may entail centering distributions of MPT parameter on specific values. For example, for source monitoring tasks influenced by expectations based on prior knowledge as in the first case study Bell et al. (2015), one might assume guessing to be expectancy-based around .60 or higher. Similarly, the choice of the prior distribution for the source memory and item memory parameters can be influenced by the paradigm expertise of the MPT modeler. In cases where the researcher possesses valuable insights and expectations regarding these parameters, it is possible to replace the prior distribution proposed here with one that favors the anticipated values.

As a refinement to current practices, we advise modelers to justify their priors theoretically and to visualize the prior predictions from their models. As the priors may influence the Bayes factors and hence conclusions drawn from the data, the informative priors should be developed prior to data collection and mentioned in the preregistration. Since visualizations of the prior predictions contribute to the understanding of the model, we advise MPT modelers to present them to the readers of their manuscript at least as an (online) appendix.

### Testing theories directly using ordinal constrained inference

In addition to specifying the statistical model within an experimental condition, we also discussed how to test expected effects on model parameters across experimental conditions using Bayes factors. Research questions in MPT research are often characterized as ordinal expectations in the form of ordinal and disordinal interactions, but so far it has been challenging to evaluate them. Commonly used methods to test ordinal expectations require the reparametrization of the MPT parameters (Knapp & Batchelder, 2004), are mainly applicable for non-hierarchical models, and are not suited to test these expectations directly.

As a further refinement to current practices, we therefore suggested computing Bayes factors using Savage–Dickey and the unconditional encompassing method (Gelfand et al., 1992; Klugkist et al., 2005; Sedransk et al., 1985). The methods discussed here have three considerable advantages over the current methods, as (1) they do not require the reparametrization of MPT parameters in order to represent interaction effects, (2) they are suited for hierarchical models, thus taking into account participant heterogeneity and hierarchical shrinkage, and (3) they are able to test theories directly. Furthermore, the methods allow researchers to test a wide variety of research questions, including the assessment of individual differences. Finally, the unconditional encompassing method relies simply on counting instances from the prior and posterior distribution, and thus is intuitive and simple to use.

However, the unconditional encompassing method comes with some limitations. First, testing parameters without reparameterization bears the risk that certain effects could be magnified or reduced depending on the scale on which they are tested. This problem is especially pronounced when the parameters are located on the boundaries of the distributions. Here we decided to test the effects on the probability scale to be consistent with the theoretical prediction. As an additional sensitivity analysis, we repeated the analysis on the probit scale, which did not change our conclusions.

Furthermore, we assessed the computational stability of the Bayes factor estimates through repeated computations. The robustness of the Bayes factor depends largely on whether enough prior and posterior samples in agreement with the constraint can be drawn from the encompassing model in order to estimate the proportion of restricted parameter space reliably. That is, if the number of restricted parameters is large or the restricted parameter space decreases (e.g., if the data suggests extreme evidence against the restricted model) the Bayes factor results become unreliable (e.g., Sarafoglou et al. 2023). This issue might, for instance, occur for the assessment of individual differences in the previous section. As a first remedy, more samples can be drawn from the encompassing model to stabilize the Bayes factor estimates, but ultimately more efficient alternatives must be developed. Alternatives to the unconditional encompassing approach are the conditional encompassing method (Mulder et al., 2009) and the recently developed bridge sampling method to evaluate restricted models (Sarafoglou et al., 2023; Gronau et al., 2020, 2019). These methods have already been applied to test order constraints in multinomial models, but not yet to test these restrictions on the class of (hierarchical) MPT models (e.g., Heck and Davis-Stober, 2019; Sarafoglou et al., 2023). A user-friendly implementation of these methods would be a key asset for Bayesian MPT modeling.

### Conclusion

Although prior specification is often considered a nuisance in Bayesian modeling, it offers MPT modelers the opportunity to make model evaluation a complete test of the theory. Heck et al.’s work has made it possible for many researchers to apply Bayesian MPT modeling to their data. Nevertheless, we believe that researchers are tempted to rely entirely on the default settings of TreeBUGS. We hope that we have succeeded in drawing attention to the potential problems with diffuse Matzke–Klauer priors, which were developed as general priors for the MPT model class but not with a specific MPT model in mind, and encouraged researchers to give the specification of priors for their particular MPT model the attention it deserves.

Even though the focus of this article was on source memory models, our arguments extend to all MPT research. MPT research is characterized by complex experimental designs and predictions often describe ordinal relations of parameters that span multiple factor levels. In combination with informative priors, the specification of ordinal expectation brings MPT modelers closer to quantitatively describing and testing their theory to the fullest extent possible.

### Supplementary Information

Below is the link to the electronic supplementary material.

Supplementary file 1 (tex 21 KB)

## Figures and Tables

**Fig. 11:**
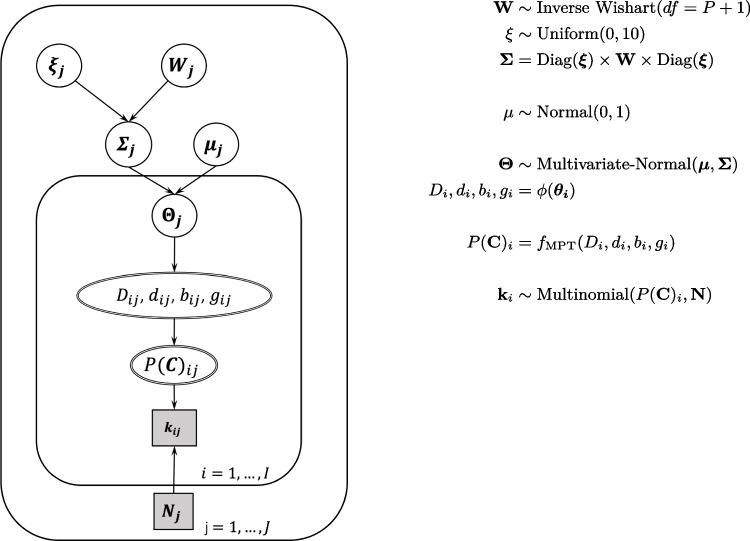
Graphical model for the default 2HTSM model as implemented in TreeBUGS with two within-subjects conditions and *J* between-subjects conditions. Index *I* refers to the total number of participants within each between-subjects condition

**Fig. 12:**
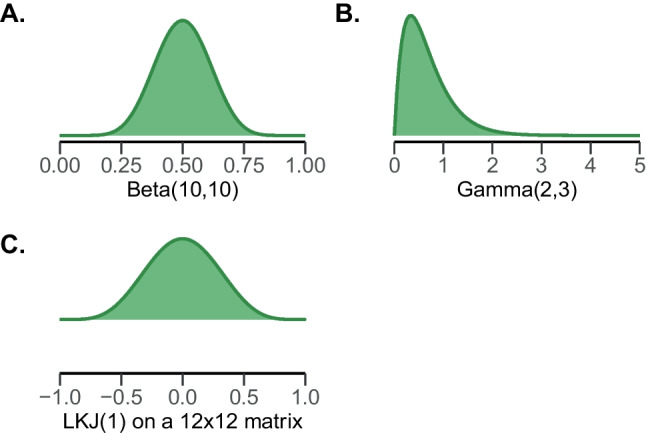
informative prior distributions for guessing parameters (**A.**), the standard deviations across participants (**B.**), and marginal correlation for LKJ(1) prior on matrices with 12 dimensions (**C.**)

**Fig. 13:**
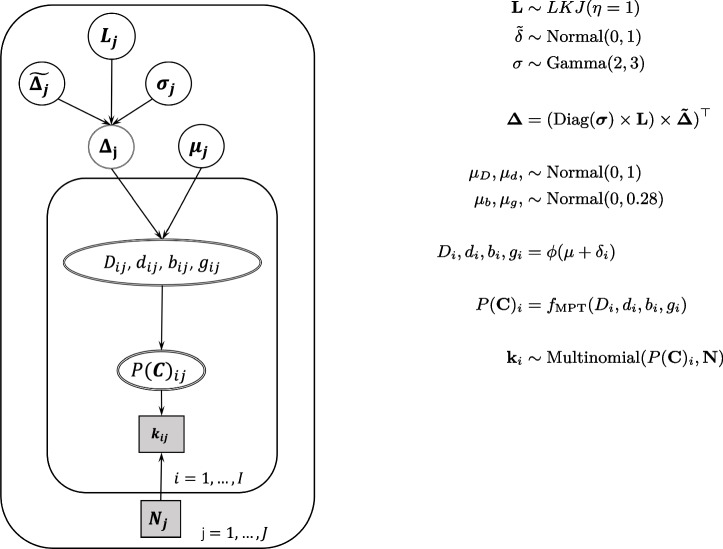
Graphical model for 2HTSM model with informative priors with two within-subjects conditions and *J* between-subjects conditions. Index *I* refers to the total number of participants within each between-subjects condition

## Appendix

### Model specifications

To model individual-level differences in the latent-trait approach responses are aggregated over items so that we receive a vector of category frequencies for each participant *i* ($i = 1, \cdots , I$) in each between-subjects experimental condition *j* ($j = 1, \cdots , J$). For convenience, we will drop subscript *j* in the following paragraphs. The individual-level MPT parameters are denoted as $D_{i}, d_{i}, b_{i}$ and $g_{i}$. The function $f_\text {MPT}$ that encodes the model equation translates the parameters into category probabilities $P({\textbf {C}})_i$, where a category corresponds, for instance, to the probability to answer “A” given that the correct source was Source A, $P(\text {``A''} \mid \text {Source A})_i$. Differences between the default 2HTSM model proposed in Matzke et al. (2015) and the informative model used in this manuscript lies in the prior distributions for the group-level parameters, that is, the prior distribution for the guessing parameter (panel A in Fig. 12), the prior distribution on the standard deviations across participants (panel B), and the marginal correlation across participants (panel C and D).

### Default prior distributions

The graphical model using default prior distributions is illustrated in Fig. 11. In this model, the vector containing the individual-level MPT parameters is probit-transformed into the vector $\varvec{\theta _i}$. The vectors over all participants are then combined in a matrix $\varvec{\Theta }$ and assigned a multivariate normal distribution as prior distribution with mean vector $\varvec{\mu }$ and a covariance matrix $\varvec{\Sigma }$. As proposed in Heck et al. (2018) and Matzke et al. (2015), the group-level MPT parameters are assumed to be independent so that each element in $\varvec{\mu }$ is assigned an independent standard normal distribution. The covariance matrix is composed of a vector containing scaling parameters $\varvec{\xi }$ and a matrix ${\textbf {W}}$. In the original work by Matzke et al. (2015), each element in $\varvec{\xi }$ is assigned a uniform distribution ranging from 0 to 100. In TreeBUGS, Heck et al. (2018) assign as default a uniform distribution ranging from 0 to 10 which limits the individual variability compared to priors proposed by Matzke et al. (2015). Lastly, the matrix ${\textbf {W}}$ is assigned an inverse-Wishart as prior distribution with degrees of freedom $df = P + 1$, where *P* refers to the number of free participant parameters.

### informative prior distributions

The graphical model using informative prior distributions is illustrated in Fig. 13. The adaptations from the classical latent-trait model are based on recommendations of Stan Development Team (2022) and Barnard et al. (2000) and implemented in Singmann (2019). In this model, the probit-transformed individual-level MPT parameters are assumed to be determined by the mean vector $\varvec{\mu }$ featuring the group-level MPT parameters and by a matrix $\varvec{\Delta }$ containing participants’ individual deviations around the group mean. As in the default model, we assume that the elements in $\varvec{\mu }$ are independent. We assign the group-level memory parameters a standard normal distribution as prior. The guessing parameters are assigned a $\text {Normal}(0, 0.28)$ distribution as prior which centers the guessing parameters around the nominal guessing level. The individual deviation matrix $\varvec{\Delta }$ is obtained by drawing from a multivariate normal distribution with means $\mu = 0$. The variance-covariance matrix of this distribution is decomposed into a vector of standard deviations $\varvec{\sigma }$ and a Cholesky-factorized correlation matrix ${\textbf {L}}$, which are used to scale a matrix of standardized deviation $\varvec{\tilde{\Delta }}$ from a standard normal distribution. To constrain individual variability and facilitate hierarchical shrinkage, we assigned each element in $\varvec{\sigma }$ a $\text {Gamma}(2, 3)$ distribution, favoring small standard deviations over large ones. We further assign an LKJ prior distribution with shape $\eta = 1$ to the correlation matrix ${\textbf {L}}$. This yields a marginal distribution favoring correlations among parameters in the range $[-0.5, 0.5]$ over more extreme correlations, see Fig. 12 panels C and D.

## Footnotes

1 Certain literature combines subjective and objective Bayesian approaches, acknowledging that not all parameters require informative prior distributions. For instance, Voormann et al. (2021) and Gronau et al. (2019) exclusively assign informative priors to test-relevant parameters and non-informative priors to the remaining parameters.

2 Even though the U-shape on the individual-level parameters displayed in Fig. 2 may resemble Jeffreys’ $\text {Beta}(\frac{1}{2}, \frac{1}{2})$ prior, they are not the equivalent. Jeffreys’ prior on probability parameters was proposed for single-parameter models and which do not readily generalize to complex hierarchical MPT models. The U-shape on the individual-level parameters may instead introduce some level of bias or add information that is not appropriate for the specific modeling problem.

3 Testing ordinal constraints is still possible for individual-level parameters, even if the Savage–Dickey Bayes factor cannot be applied.

4 A robustness analysis in the probit space did not change the conclusions drawn in our case study.

5 This disordinal interaction was an exploratory finding in experiment 1 which the authors then tested on data from Experiment 2. For simplicity, we test this disordinal prediction on the data from both experiments.

6 Model adequacy in terms of quantitative measures of model fit was also confirmed for the model with Matzke-Klauer priors.

## References

[CR1] Aczel, B., Hoekstra, R., Gelman, A., Wagenmakers, E.-J., Klugkist, I. G., Rouder, J. N., Vandekerckhove, J., Lee, M. D., Morey, R. D., Vanpaemel, W., Dienes, Z., & van Ravenzwaaij, D. (2020). Discussion points for Bayesian inference. *Nature Human Behaviour,**4*, 561–563. 31988442 10.1038/s41562-019-0807-z

[CR2] Barnard, J., McCulloch, R., & Meng, X.-L. (2000). Modeling covariance matrices in terms of standard deviations and correlations, with application to shrinkage. *Statistica Sinica,**10*, 1281–1311.

[CR3] Batchelder, W., & Riefer, D. M. (1999). Theoretical and empirical review of multinomial process tree modeling. *Psychonomic Bulletin & Reviewy,**6*, 57–86. 10.3758/bf03210812 12199315

[CR4] Batchelder, W., & Riefer, D. M. (1990). Multinomial processing models of source monitoring. *Psychological Review,**97*, 548–564.

[CR5] Bayen, U. J., Murnane, K., & Erdfelder, E. (1996). Source discrimination, item detection, and multinomial models of source monitoring. *Journal of Experimental Psychology: Learning, Memory, and Cognition,**22*, 197–215.

[CR6] Bell, R., Buchner, A., Kroneisen, M., & Giang, T. (2012). On the flexibility of social source memory: A test of the emotional incongruity hypothesis. *Journal of Experimental Psychology: Learning, Memory, and Cognition,**38*, 1512–1529. 22545603 10.1037/a0028219

[CR7] Bell, R., Mieth, L., & Buchner, A. (2015). Appearance-based first impressions and person memory. *Journal of Experimental Psychology: Learning, Memory, and Cognition,**41*, 456–472. 24999709 10.1037/xlm0000034

[CR8] Berger, J. (2004). The case for objective Bayesian analysis. *Bayesian Analysis,**1*, 1–17.

[CR9] Bernardo, J. M. (1979). Reference posterior distributions for Bayesian inference. *Journal of the Royal Statistical Society: Series B (Methodological),**41*, 113–128.

[CR10] Chechile, R. A. (2009). Pooling data versus averaging model fits for some prototypical multinomial processing tree models. *Journal of Mathematical Psychology,**53*, 562–576.

[CR11] Dickey, J. M. (1971). The weighted likelihood ratio, linear hypotheses on normal location parameters. *The Annals of Mathematical Statistics,**42*, 204–223.

[CR12] Dickey, J. M., & Lientz, B. (1970). The weighted likelihood ratio, sharp hypotheses about chances, the order of a Markov chain. *The Annals of Mathematical Statistics,**41*, 214–226.

[CR13] Dienes, Z. (2011). Bayesian versus orthodox statistics: Which side are you on? *Perspectives on Psychological Science,**6*, 274–290. 26168518 10.1177/1745691611406920

[CR14] Erdfelder, E., Auer, T.-S., Hilbig, B. E., Aßfalg, A., Moshagen, M., & Nadarevic, L. (2009). Multinomial processing tree models: Areview of the literature. *Zeitschrift für Psychologie/ Journal of Psychology,**217*, 108–124.

[CR15] Gabry, J., Simpson, D., Vehtari, A., Betancourt, M., & Gelman, A. (2019). Visualization in Bayesian workflow. *Journal of the Royal Statistical Society: Series A (Statistics in Society),**182*, 389–402.

[CR16] Gelfand, A. E., Smith, A. F., & Lee, T.-M. (1992). Bayesian analysis of constrained parameter and truncated data problems using gibbs sampling. *Journal of the American Statistical Association,**87*, 523–532.

[CR17] Gronau, Q. F., Singmann, H., & Wagenmakers, E.-J. (2020). Bridgesampling: An R package for estimating normalizing constants. *Journal of Statistical Software, Articles,**92*(10), 1–29.

[CR18] Gronau, Q. F., Wagenmakers, E.-J., Heck, D. W., & Matzke, D. (2019). A simple method for comparing complex models: Bayesian model comparison for hierarchical multinomial processing tree models using Warp-III bridge sampling. *Psychometrika,**84*, 261–284. 30483923 10.1007/s11336-018-9648-3 PMC6684497

[CR19] Gronau, Q., Raj, A., Wagenmakers, E.-J., et al. (2021). Informed Bayesian inference for the A/B test. *Journal of Statistical Software,**100*, 1–39.

[CR20] Gu, X., Mulder, J., & Hoijtink, H. (2018). Approximated adjusted fractional Bayes factors: A general method for testing informative hypotheses. *British Journal of Mathematical and Statistical Psychology,**71*, 229–261. 28857129 10.1111/bmsp.12110

[CR21] Haaf, J. M., & Rouder, J. N. (2017). Developing constraint in Bayesian mixed models. *Psychological Methods,**22*, 779–798. 29265850 10.1037/met0000156

[CR22] Haaf, J. M., & Rouder, J. N. (2019). Some do and some don’t? accounting for variability of individual difference structures. *Psychonomic Bulletin & Review,**26*, 772–789. 30251148 10.3758/s13423-018-1522-x

[CR23] Heck, D. W. (2019). A caveat on the Savage-Dickey density ratio: The case of computing Bayes factors for regression parameters. *British Journal of Mathematical and Statistical Psychology,**72*, 316–333. 30451277 10.1111/bmsp.12150

[CR24] Heck, D. W., Arnold, N. R., & Arnold, D. (2018). Treebugs: An R package for hierarchical multinomial-processing-tree modeling. *Behavior Research Methods,**50*, 264–284. 28374146 10.3758/s13428-017-0869-7 PMC5809562

[CR25] Heck, D. W., & Davis-Stober, C. P. (2019). Multinomial models with linear inequality constraints: Overview and improvements of computational methods for Bayesian inference. *Journal of Mathematical Psychology,**91*, 70–87. 30956351 10.1016/j.jmp.2019.03.004 PMC6448806

[CR26] Heck, D. W., & Erdfelder, E. (2019). Maximizing the expected information gain of cognitive modeling via design optimization. *Computational Brain & Behavior,**2*, 202–209.

[CR27] Heck, D. W., & Wagenmakers, E.-J. (2016). Adjusted priors for Bayes factors involving reparameterized order constraints. *Journal of Mathematical Psychology,**73*, 110–116.

[CR28] Holcombe, A. O., Kovacs, M., Aust, F., & Aczel, B. (2020). Documenting contributions to scholarly articles using CRediT and tenzing. *PLoS One,**15*, e0244611. 33383578 10.1371/journal.pone.0244611 PMC7775117

[CR29] Jaynes, E. T. (2016). Discrete prior probabilities: The entropy principle. In G. Larry Bretthorst (Ed.), *Probability theory: The logic of science* (pp. 343–371). American Psychological Association.

[CR30] Jeffreys, H. (1935). Some tests of significance, treated by the theory of probability. *Proceedings of the Cambridge Philosophy Society,**31*, 203–222.

[CR31] Johnson, M. K., Hashtroudi, S., & Lindsay, D. S. (1993). Source monitoring. *Psychological Bulletin,**114*, 3–28. 8346328 10.1037/0033-2909.114.1.3

[CR32] Kass, R. E., & Raftery, A. E. (1995). Bayes factors. *Journal of the American Statistical Association,**90*, 773–795.

[CR33] Keynes, J. M. (1921). The principle of indifference. A treatise on probability (1st ed., pp. 41– 64). Dover Publications Inc.

[CR34] Klauer, K. C. (2010). Hierarchical multinomial processing tree models: A latent-trait approach. *Psychometrika,**75*, 70–98.

[CR35] Klauer, K. C., Singmann, H., & Kellen, D. (2015). Parametric order constraints in multinomial processing tree models: An extension of Knapp and Batchelder (2004). *Journal of Mathematical Psychology,**64*, 215–229.

[CR36] Klugkist, I., Kato, B., & Hoijtink, H. (2005). Bayesian model selection using encompassing priors. *Statistica Neerlandica,**59*, 57–69.

[CR37] Knapp, B. R., & Batchelder, W. (2004). Representing parametric order constraints in multitrial applications of multinomial processing tree models. *Journal of Mathematical Psychology,**48*, 215–229.

[CR38] Kooperberg, C. (2020). Logspline: Routines for logspline density estimation [R package version 2.1.16]. https://CRAN.R-project.org/package=logspline

[CR39] Kuhlmann, B. G., Erdfelder, E., & Moshagen, M. (2019). Testing interactions in multinomial processing tree models. *Frontiers in psychology,**10*, 2364. 31736818 10.3389/fpsyg.2019.02364 PMC6837999

[CR40] Lee, M. D. (2018). Bayesian methods in cognitive modeling. In J. T. Wixted & E.-J. Wagenmakers (Eds.), The Stevens’ handbook of experimental psychology and cognitive neuroscience: Vol. 5 Methodology (4th ed., pp. 37–84).

[CR41] Lee, M. D., & Vanpaemel, W. (2018). Determining informative priors for cognitive models. *Psychonomic Bulletin & Review,**25*, 114–127. 28194721 10.3758/s13423-017-1238-3

[CR42] Lee, M. D., Criss, A. H., Devezer, B., Donkin, C., Etz, A., Leite, F. P., Matzke, D., Rouder, J. N., Trueblood, J. S., White, C. N., & Vandekerckhove, J. (2019). Robust modeling in cognitive science. *Computational Brain & Behavior,**2*, 141–153.

[CR43] Martin, S. R. (2021). Informative priors for correlation matrices: An easy approach. http://srmart.in/informative-priors-for-correlation-matrices-an-easy-approach/

[CR44] Matzke, D., Dolan, C. V., Batchelder, W., & Wagenmakers, E.-J. (2015). Bayesian estimation of multinomial processing tree models with heterogeneity in participants and items. *Psychometrika,**80*, 205–235. 24277381 10.1007/s11336-013-9374-9

[CR45] Miller, J., & Schwarz, W. (2018). Implications of individual differences in on-average null effects. *Journal of Experimental Psychology: General,**147*, 377–397. 29058941 10.1037/xge0000367

[CR46] Moreno, E., & Pericchi, L. R. (2014). Intrinsic priors for objective Bayesian model selection. In I. Jeliazkov & D. J. Poirier (Eds.), *Bayesian model comparison (advances in econometrics)* (pp. 279–300). Emerald Group Publishing Limited.

[CR47] Morey, R. D., Rouder, J. N., Pratte, M. S., & Speckman, P. L. (2011). Using MCMC chain outputs to efficiently estimate Bayes factors. *Journal of Mathematical Psychology,**55*, 368–378.

[CR48] Moshagen, M. (2010). MultiTree: A computer program for the analysis of multinomial processing tree models. *Behaviour Research Methods,**42*, 42–54. 10.3758/BRM.42.1.42 20160285

[CR49] Mulder, J., Klugkist, I., van de Schoot, R., Meeus, W. H. J., Selfhout, M., & Hoijtink, H. (2009). Bayesian model selection of informative hypotheses for repeated measurements. *Journal of Mathematical Psychology,**53*, 530–546.

[CR50] Myung, I. J., & Pitt, M. A. (1997). Applying Occam’s razor in modeling cognition: A Bayesian approach. *Psychonomic Bulletin & Review,**4*, 79–95.

[CR51] O’Hagan, A. (1995). Fractional Bayes factors for model comparison. *Journal of the Royal Statistical Society: Series B (Methodological),**57*, 99–118.

[CR52] O’Hagan, A., Buck, C. E., Daneshkhah, A., Eiser, J. R., Garthwaite, P. H., Jenkinson, D. J., Oakley, J. E., & Rakow, T. (2006). Uncertain judgements: Eliciting experts’ probabilities.

[CR53] Riefer, D. M., & Batchelder, W. (1988). Multinomial modeling and the measurement of cognitive processes. *Psychological Review,**95*, 318–339.

[CR54] Rouder, J. N., & Lu, J. (2005). An introduction to Bayesian hierarchical models with an application in the theory of signal detection. *Psychonomic Bulletin & Review,**12*, 573–604. 16447374 10.3758/bf03196750

[CR55] Rouder, J. N., Lu, J., Morey, R. D., Sun, D., & Speckman, P. L. (2008). A hierarchical process-dissociation model. *Journal of Experimental Psychology: General,**137*(2), 370–389. 18473664 10.1037/0096-3445.137.2.370

[CR56] Rouder, J. N., Morey, R. D., & Pratte, M. S. (2017). Bayesian hierarchical models of cognition. In W. Batchelder, H. Colonius, E. N. Dzhafarov, & J. Myung (Eds.), *New handbook of mathematical psychology: Foundations and methodology* (pp. 504–551). Cambridge University Press.

[CR57] Rouder, J. N., Morey, R. D., & Wagenmakers, E.-J. (2016). The interplay between subjectivity, statistical practice, and psychological science. *Collabra,**2*, 6.

[CR58] Sarafoglou, A., Haaf, J. M., Ly, A., Gronau, Q. F., Wagenmakers, E.-J., & Marsman, M. (2023). Evaluating multinomial order restrictions with bridge sampling. *Psychological Methods,**28*, 322–338. 34914473 10.1037/met0000411

[CR59] Sarafoglou, A., Aust, F., Marsman, M., Wagenmakers, E.-J., & Haaf, J. M. (2023). Multibridge: An R package to evaluate informed hypotheses in binomial and multinomial models. *Behaviour Research Methods*, *55*, 4343–4368. 10.3758/s13428-022-02020-1 PMC10700431 37277644

[CR60] Schad, D. J., Betancourt, M., & Vasishth, S. (2021). Toward a principled Bayesian workflow in cognitive science. *Psychological Methods,**26*(1), 103–126. 32551748 10.1037/met0000275

[CR61] Schmidt, O., Erdfelder, E., & Heck, D. W. (2022). Tutorial on multinomial processing tree modeling: How to develop, test, and extend MPT models. Manuscript submitted for publication. 10.31234/osf.io/gh8md

[CR62] Sedransk, J., Monahan, J., & Chiu, H. (1985). Bayesian estimation of finite population parameters in categorical data models incorporating order restrictions. Journal of the Royal Statistical Society. *Series B (Methodological),**47*, 519–527.

[CR63] Shannon, C. E. (1948). A mathematical theory of communication. *The Bell System Technical Journal,**27*, 379–423.

[CR64] Singmann, H. (2019). Bayesian cognitive modeling: MPT case studies.

[CR65] Sinharay, S., & Stern, H. S. (2002). On the sensitivity of Bayes factors to the prior distributions. *The American Statistician,**56*, 196–201.

[CR66] Smith, J. B., & Batchelder, W. (2010). Beta-MPT: Multinomial processing tree models for addressing individual differences. *Journal of Mathematical Psychology,**54*, 167–183. 10.1016/j.jmp.2010.02.001 PMC2875838 20514139

[CR67] Smith, J. B., & Batchelder, W. (2008). Assessing individual differences in categorical data. *Psychonomic Bulletin & Review,**15*, 713–731. 18792498 10.3758/pbr.15.4.713

[CR68] Stan Development Team. (2022). Multivariate priors for hierarchical models. Stan user’s guide, version 2.29.0 (pp. 35–43). http://mc-stan.org/

[CR69] Stefan, A. M., Evans, N. J., & Wagenmakers, E.-J. (2020). Practical challenges and methodological flexibility in prior elicitation. *Psychological Methods*. 10.1037/met0000354 32940511

[CR70] Stefan, A. M., Katsimpokis, D., Gronau, Q. F., & Wagenmakers, E.-J. (2022). Expert agreement in prior elicitation and its effects on Bayesian inference. *Psychonomic Bulletin & Review,**29*, 1776–1794. 35378671 10.3758/s13423-022-02074-4 PMC9568464

[CR71] Stone, C. J., Hansen, M. H., Kooperberg, C., & Truong, Y. K. (1997). Polynomial splines and their tensor products in extended linear modeling (with discussion). *The Annals of Statistics,**25*, 1371–1470.

[CR72] Symeonidou, N., & Kuhlmann, B. G. (2021). A novel paradigm to assess storage of sources in memory: The source recognition test with reinstatement. Memory, 1–17. 10.1080/09658211.2021.1910310 33847239

[CR73] Thiele, J. E., Haaf, J. M., & Rouder, J. N. (2017). Is there variation across individuals in processing? Bayesian analysis for systems factorial technology. *Journal of Mathematical Psychology,**81*, 40–54.

[CR74] Tran, N.-H., Van Maanen, L., Heathcote, A., & Matzke, D. (2021). Systematic parameter reviews in cognitive modeling: Towards a robust and cumulative characterization of psychological processes in the diffusion decision model. *Frontiers in Psychology,**11*, 608287. 10.3389/fpsyg.2020.608287 PMC7874054 33584443

[CR75] van den Bergh, D., Wagenmakers, E.-J., & Aust, F. (2022). Bayesian repeated-measures ANOVA: An updated methodology implemented in JASP. https://psyarxiv.com/fb8zn/.

[CR76] Vanpaemel, W. (2010). Prior sensitivity in theory testing: An apologia for the Bayes factor. *Journal of Mathematical Psychology,**54*, 491–498.

[CR77] Vanpaemel, W., & Lee, M. D. (2012). Using priors to formalize theory: Optimal attention and the generalized context model. *Psychonomic Bulletin & Review,**19*, 1047–1056. 22869335 10.3758/s13423-012-0300-4

[CR78] Verdinelli, I., & Wasserman, L. (1995). Computing Bayes factors using a generalization of the Savage-Dickey density ratio. *Journal of the American Statistical Association,**90*, 614–618.

[CR79] Voormann, A., Spektor, M. S., & Klauer, K. C. (2021). The simultaneous recognition of multiple words: A process analysis. *Memory & Cognition,**49*, 787–802. 33834382 10.3758/s13421-020-01082-w PMC8081710

[CR80] Wagenmakers, E.-J., Lodewyckx, T., Kuriyal, H., & Grasman, R. (2010). Bayesian hypothesis testing for psychologists: A tutorial on the Savage-Dickey method. *Cognitive Psychology,**60*, 158–189. 20064637 10.1016/j.cogpsych.2009.12.001

[CR81] Wagenmakers, E.-J., Sarafoglou, A., Aarts, S., Albers, C., Algermissen, J., Bahnık, Š, van Dongen, N., Hoekstra, R., Moreau, D., van Ravenzwaaij, D., Sluga, A., Stanke, F., Tendeiro, J., & Aczel, B. (2021). Seven steps toward more transparency in statistical practice. *Nature Human Behaviour,**5*, 1473–1480. 34764461 10.1038/s41562-021-01211-8

[CR82] Webb, M. R., & Lee, M. D. (2004). Modeling individual differences in category learning. *Proceedings of the Annual Meeting of the Cognitive Science Society,**26*(26), 1440–1445.

[CR83] Wetzels, R., Grasman, R., & Wagenmakers, E.-J. (2010). An encompassing prior generalization of the Savage-Dickey density ratio. *Computational Statistics & Data Analysis,**54*, 2094–2102.

